# From death to birth: how osteocyte death promotes osteoclast formation

**DOI:** 10.3389/fimmu.2025.1551542

**Published:** 2025-03-17

**Authors:** Weijie Zhao, Jiale Qian, Ji Li, Tian Su, Xiaozhong Deng, Yonghua Fu, Xuelong Liang, Hongwang Cui

**Affiliations:** ^1^ Key Laboratory of Emergency and Trauma of Ministry of Education, Department of Emergency Surgery, Key Laboratory of Hainan Trauma and Disaster Rescue, The First Affiliated Hospital, Hainan Medical University, Haikou, China; ^2^ Key Laboratory of Emergency and Trauma, Ministry of Education, Key Laboratory of Haikou Trauma, Key Laboratory of Hainan Trauma and Disaster Rescue, The First Affiliated Hospital of Hainan Medical University, Hainan Medical University, Haikou, China; ^3^ Engineering Research Center for Hainan Bio-Smart Materials and Bio-Medical Devices, Key Laboratory of Hainan Functional Materials and Molecular Imaging, College of Emergency and Trauma, College of pharmacy, Hainan Medical University, Haikou, China; ^4^ Department of Pain Treatment, Nanxi Shan Hospital of Guangxi Zhuang Autonomous Region, Guilin, China; ^5^ Department of Hand and Foot Microsurgery, The Second Affiliated Hospital of Hainan Medical University, Haikou, China; ^6^ The First School of Clinical Medicine, Southern Medical University, Guangzhou, China

**Keywords:** osteocyte, necrosis, apoptosis, necroptosis, ferroptosis, pyroptosis, osteoclast, osteoimmunology

## Abstract

Bone remodeling is a dynamic and continuous process involving three components: bone formation mediated by osteoblasts, bone resorption mediated by osteoclasts, and bone formation-resorption balancing regulated by osteocytes. Excessive osteocyte death is found in various bone diseases, such as postmenopausal osteoporosis (PMOP), and osteoclasts are found increased and activated at osteocyte death sites. Currently, apart from apoptosis and necrosis as previously established, more forms of cell death are reported, including necroptosis, ferroptosis and pyroptosis. These forms of cell death play important role in the development of inflammatory diseases and bone diseases. Increasing studies have revealed that various forms of osteocyte death promote osteoclast formation via different mechanism, including actively secreting pro-inflammatory and pro-osteoclastogenic cytokines, such as tumor necrosis factor alpha (TNF-α) and receptor activator of nuclear factor-kappa B ligand (RANKL), or passively releasing pro-inflammatory damage associated molecule patterns (DAMPs), such as high mobility group box 1 (HMGB1). This review summarizes the established and potential mechanisms by which various forms of osteocyte death regulate osteoclast formation, aiming to provide better understanding of bone disease development and therapeutic target.

## 1 Introduction

Bone remodeling is a dynamic and continuous process that ensures skeletal integrity by balancing bone formation and resorption. This process is essential for maintaining bone homeostasis, repairing microdamage, and adapting to mechanical forces. Bone remodeling involves three major cell types: osteoclasts, which mediate bone resorption; osteoblasts, which mediate bone formation; and osteocytes, which are embedded within the bone matrix and serve as key regulators of the remodeling process (1–3). Osteocytes, accounting for over 90% of bone cells, act as the important mechano-sensors and orchestrators of bone remodeling by balancing the activities of osteoclasts and osteoblasts (4, 5). Unbalance between osteoclast-mediated bone resorption and osteoblast-mediated bone formation will lead to abnormal bone remodeling, which, if the unbalance prolongs, will further lead to various inflammatory bone diseases, such as osteoporosis, periodontal disease and rheumatoid arthritis (6). These diseases are partially characterized by excess osteoclastic activity. Osteoclasts differentiate from mononuclear/macrophages (7). This process is regulated by various signals from immune cells and bone cells. These interaction between bone cells and immune cells are recently referred as “osteoimmunology” (8). Among these osteoimmune signals, receptor activator of nuclear factor-kappa B ligand (RANKL) is the predominant cytokine and is crucial in osteoclast formation (9). Studies have reported that osteocytes are an essential source of RANKL (10, 11), making themselves a crucial regulator of osteoclast formation and activation. Under physiological condition, osteocytes primarily exert inhibitory effects on osteoclast formation, while their death turns off the inhibition and exert pro-osteoclastogenic effect in multiple ways (12). Like death of other cell types, osteocyte death also triggers immune reaction, but in its unique way which ultimately recruits osteoclasts. Studies found that apoptotic osteocytes could promote osteoclast formation by increasing expression of pro-osteoclastogenic cytokines, such as RANKL, and releasing pro-inflammatory damage associated molecular patterns (DAMPs), such as high mobility group box 1 (HMGB1) (13, 14). In addition to apoptosis, studies have revealed novel types of cell death, such as necroptosis and ferroptosis. Osteocytes undergoing these types of cell death are reported to induce inflammation by releasing pro-inflammatory factors and DAMPs, which participate in the development of bone diseases (15, 16). Given the important role of osteocyte-osteoclast crosstalk in bone remodeling and bone diseases, it is necessary to understand whether and how osteocytes undergoing various types of cell death are involved in regulation of osteoclast formation. This review summarizes various types of cell death in osteocytes and their potential mechanism to regulate osteoclast formation, with the aim to further understand the development of bone disease and to explore potential therapeutic target.

## 2 Osteoclast formation and regulation

### 2.1 A brief overview of osteoclast formation and activation

**Figure 1:**
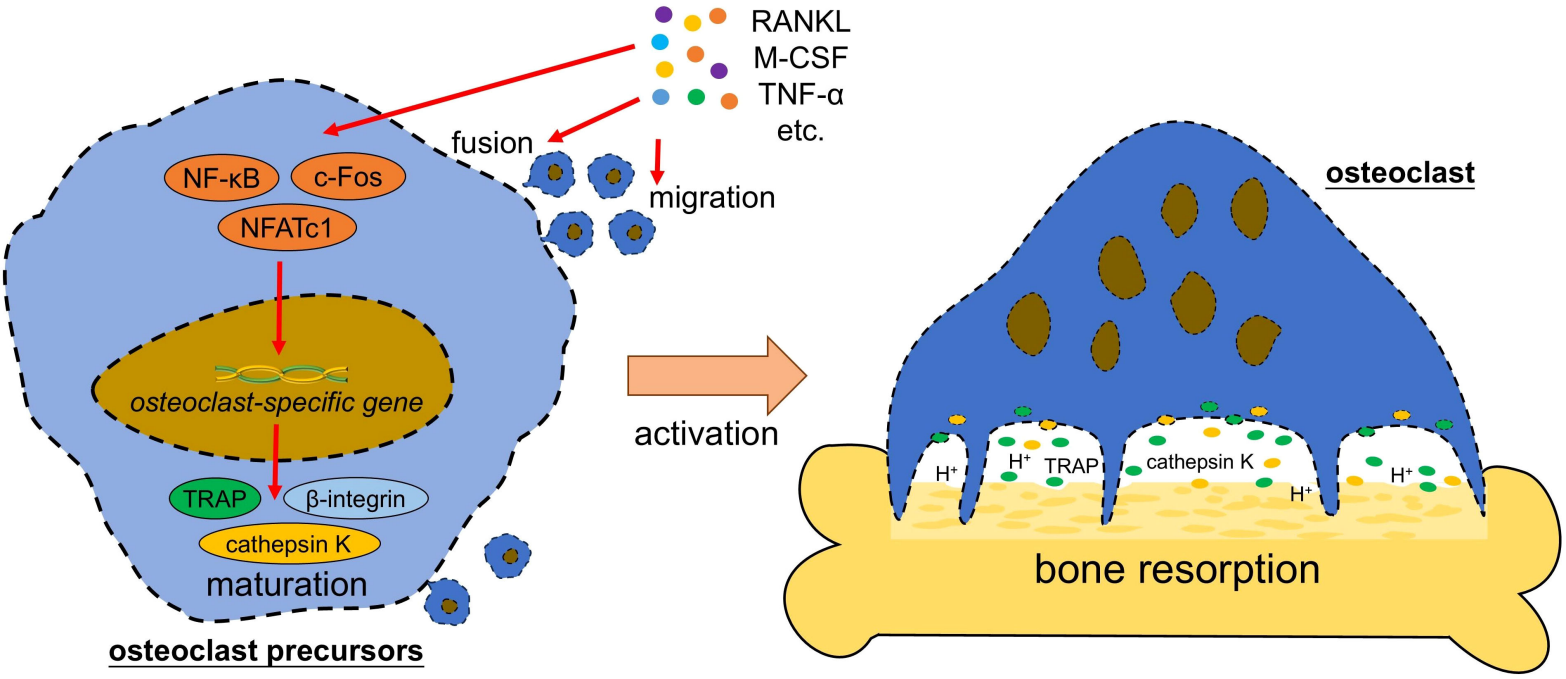
Osteoclast formation and activation. Pro-osteoclastogenic factors (such as RANKL and M-CSF) activate the transcription factors (such as NF-κB and NFATc1) in osteoclast precursor, which further trigger the expression of osteoclast-specific gene to initiate the maturation of osteoclast and activation of bone resorption. RANKL, receptor activator of nuclear factor-kappa B ligand; M-CSF, macrophage colony stimulating factor; TNF-α, tumor necrosis factor alpha; NF-κB, nuclear factor-kappa B; NFATc1, nuclear factor of activated T cells cytoplasmic 1; TRAP, tartrate-resistant acid phosphatase; H^+^, hydrogen ion.

Osteoclasts originate from mononuclear phagocytes, which comprise monocytes/macrophages and dendritic cells (DC) (7, 17). These mononuclear cells with potential to form osteoclasts are called osteoclast precursors (OCPs) and located in multiple tissue, including bone marrow, spleen, thymus, or peripheral blood (18). The differentiation of OCPs to mature osteoclasts requires macrophage colony stimulating factor (M-CSF) and RANKL, which induce the expression of osteoclast-specific genes, including those encoding tartrate-resistant acid phosphatase (TRAP), β-integrin and cathepsin K (**Figure 1**). These are key proteins that lead to the maturation of osteoclasts (6). Mature osteoclast is the multinucleated cell with at least three nuclei. Its cytoplasm contains large amounts of vesicles that are filled with TRAP and cathepsin K. When activated by extracellular signals of bone remodeling, osteoclast undergoes polarization and reconstruction via the rearrangements of actin cytoskeleton, to form a sealed compartment between the bone surface and basal membrane of osteoclast. Then acids and lysosomal enzymes (TRAP, cathepsin K, etc.) are secreted into this sealed compartment to initiate bone resorption (19).

### 2.2 Signal molecules from osteocytes and immune cells that promote osteoclast formation

Osteoclast formation is triggered by various signal molecules from osteocytes and immune cells, such as RANKL and TNF-α (**Table 1**). Some of these signal molecules are recognized as DAMPs, which refer to intracellular contents released from damaged osteocytes into extracellular matrix, such as HMGB1 and adenosine triphosphate (ATP). DAMPs can interact with downstream pattern recognition receptors (PRRs), including Toll-like receptors (TLRs) and C-type lectin receptors (CLRs), and cause the aseptic inflammatory responses (20–23). These signal molecules exert directly or indirectly regulating effects on osteoclasts in an interacting and complicated manner, and they are to be introduced as follow.

**Table 1:** Signal molecules from osteocytes and immune cells that promote osteoclast formation.

Signal molecule	Source	Pro-osteoclastogenic effects
RANKL	Viable/apoptotic/ferroptotic Ocy; OB, BC, TC, etc.	Activating NFATc1 to initiate the expression of osteoclast-related gene in OCPs
TNF-α	Apoptotic/ferroptotic Ocy; Mφ, etc.	Increasing expression of RANKL, IL-1β, etc; Mediating the switching of M2 to M1 macrophages; Directly act on OCPs to promote osteoclastogenesis
IL-1β	Viable/apoptotic/ferroptotic/pyroptotic Ocy; Dendritic cells, monocytes, TC, Mφ, etc.	Increasing expression of RANKL; Inhibiting OPG expression; Directly act on OCPs to promote osteoclast migration, formation and activation
IL-6	Apoptotic/ferroptotic Ocy; Monocytes, Mφ, TC, BC, fibroblasts, etc.	Mediating the transmigration of OCPs from blood to bone marrow; Increasing expression of RANKL
Sclerostin	Ocy, etc.	Increasing expression of RANKL; Inhibiting OPG expression;
VEGF	Viable/apoptotic Ocy; OB, chondrocytes, Mφ, etc.	Directly act on OCPs to promote osteoclastogenesis
HMGB1	Viable/apoptotic/necrotic ^pt^/necroptotic ^pt^/ferroptotic ^pt^/pyroptotic ^pt^ Ocy; Osteoclasts, OB, etc.	Increasing expression of RANKL, TNF-α, and IL-6; Directly act on OCPs to promote osteoclastogenesis
ATP	Apoptotic/damaged Ocy, etc.	(low concentration) Promoting osteoclastogenesis; (high concentration) Inhibiting osteoclastogenesis ^pt^

Ocy, osteocytes; OB, osteoblasts; BC, B lymphocyte; TC, T lymphocyte; NFATc1, nuclear factor of activated T cells cytoplasmic 1; OCPs, osteoclast precursors; Mφ, macrophages; ^pt^ Potentially.

#### 2.2.1 RANKL

Receptor activator of nuclear factor kappa-β ligand (RANKL) is a cytokine belonging to TNF superfamily. Its receptor, RANK, is a member of TNF receptor superfamily. RANKL can be expressed by various types of cells, such as osteocytes, osteoblasts, B lymphocytes, T lymphocytes, and periodontal ligament cells (11, 24–26). Among these cells, osteocytes are found to express high levels of RANKL and have greater capacity to promote osteoclastogenesis *in vitro* (11). Studies have shown that mice with gene deletion of RANKL in osteocytes exerted phenotype of osteopetrosis due to the defects in osteoclast formation, indicating that osteocytes is an essential source of RANKL (10, 11).

RANKL play crucial role in osteoclastogenesis. Binding of RANKL with RANK in OCPs, with co-stimulation of M-CSF, induces osteoclast differentiation, fusion, and activation. When binding to RANKL, RANK recruits a variety of multifunctional adaptor proteins, including the TNF receptor-associated factors (TRAFs) (18). RANK also recruits kinases, such as TGFβ activated kinase-1 (TAK1), to sequentially activate nuclear factor-kappa B (NF-κB), c-Fos, and nuclear factor of activated T cells cytoplasmic 1 (NFATc1), three transcriptional factors that are essential for osteoclastogenesis (27). NFATc1 is the master transcription factor regulating osteoclastogenesis (28, 29). Over-expression of NFATc1 results in the differentiation and fusion of OCPs without stimulation of RANKL (28, 29). In addition to the common OCPs, studies found that RANKL could induce B lymphocytes to differentiate into osteoclasts (30–32), indicating a wide range of pro-osteoclastogenic effect of RANKL.

RANKL-RANK interaction can be blocked by osteoprotegerin (OPG). OPG is a soluble decoy receptor of RANKL, with capability of preventing RANKL from binding RANK and thus inhibiting osteoclast formation and bone resorption (33). Studies have shown that genetic deletion of OPG could lead to osteoporosis in mice (34, 35), and homozygous deletion of OPG gene in human results in juvenile Paget’s disease, a disease characterized by osteopenia and fractures (36).

#### 2.2.2 TNF-α

TNF-α is a potent pro-inflammatory cytokine secreted by various cells, such as macrophages and osteocytes. TNF-α is central to the pathogenesis of inflammatory diseases, such as rheumatoid arthritis (RA) (37). Our previous studies revealed that TNF-α also plays important role in the development of postmenopausal osteoporosis (15). It has been widely reported that TNF-α plays crucial role in regulating osteoclast formation and activation (38). TNF-α exerts pro-osteoclastogenic effects in two ways: acting on osteocytes and on osteoclasts. TNF-α can increase the expression of RANKL in osteocytes. Marahleh et al. found that primary osteocytes stimulated by TNF-α showed significantly higher RANKL mRNA expression (39). They also found that mouse with TNF-α injected into the calvaria generated increased number of RANKL-positive osteocytes and osteoclasts. A further study revealed that TNF-α induced sclerostin expression in osteocytes, which further triggered RANKL expression in osteocytes, thereby enhancing osteoclastogenesis (40). On the other hand, TNF-α directly acts on OCPs to trigger osteoclastogenesis. It has been found that TNF-α could work synergistically with RANKL and M-CSF to trigger osteoclastogenesis via NF-kB and phosphatidylinositol 3 kinase (PI3k)/AKT pathway (38). Moreover, Zhao et al. found that TNF-α could mediate the M-CSF-induced switching of M2 to M1 macrophages, which have higher potential to differentiate into osteoclasts (41). While it was previously considered that RANKL was essential for TNF-α-induced osteoclastogenesis (42, 43), a recent study showed the opposite result. O’Brien et al. found that TNF-α plus interleukin-6 (IL-6) could induced osteoclast formation in mice with *Rank* deficient (44), indicating that TNF-α is able to induce osteoclastogenesis independent of RANKL. Together, TNF-α is capable of inducing osteoclastogenesis, by acting directly or indirectly on OCPs, in a manner dependent or independent on RANKL/RANK pathway.

#### 2.2.3 IL-1β

Interleukin-1β (IL-1β) is an important pro-inflammatory cytokine, normally produced by multiple cells including dendritic cells, monocytes, T lymphocytes, osteocytes and macrophages (45). IL-1β is widely involved in various inflammatory response and recently increasing studies have revealed its role in inflammatory bone diseases, such as rheumatoid arthritis and osteoporosis (46–48). It is reported that IL-1β promoted RANKL expression in MLO-Y4 osteocyte-like cells, while inhibiting OPG expression. This dual effects further increase the formation of osteoclasts (49). Moreover, IL-1β, in combination of other exogenous cytokines, such as IL-6 and TNF-α, is found to upregulates IL-1β expression in human osteocyte, forming a positive feedback loop to intensify the inflammation (50). IL-1β is also able to act on OCPs and osteoclasts directly. IL-1β promotes RANKL-induced osteoclast differentiation via the activation of NF-kB (51). IL-1β also increases the expression of C-C motif chemokine receptor 7 (CCR7), the receptor of C–C chemokine ligand 19 (CCL19) and CCL21, to promotes osteoclast migration and activation (52). Osteoclast activation requires proteolytic enzymes like plasminogen and collagenases to break down bone matrix proteins. It is reported that IL-1β could enhance the expression of these proteolytic enzymes (53). Together, these results suggest a pro-osteoclastogenic role of IL-1β.

#### 2.2.4 IL-6

IL-6 is a proinflammatory cytokine produced by various cells including monocytes, macrophages, T lymphocytes, B cells, fibroblasts (54). IL-6 is also found to derive from apoptotic osteocytes (55). IL-6 plays important roles in inflammation, autoimmunity, injury, rheumatoid arthritis, and cancer (56, 57). It is also reported to involve in bone diseases, such as osteoporosis. Tanaka et al. found that IL-6 regulated sphingosine-1-phosphate receptor (S1PR) on the OCPs. They found IL-6 upregulated the expression of S1PR2, which mediated the transmigration of OCPs from blood to bone marrow, while downregulated the expression of S1PR1, which mediated the transmigration in opposite direction (58). Besides, IL-6 inducing RANKL expression in osteoblasts and osteocytes via the Janus kinase 2 (JAK2)/signal transducer and activator of transcription 3 (STAT3) pathway, indirectly promoting osteoclast-mediated bone resorption (59–61).

#### 2.2.5 Sclerostin

Sclerostin is a glycoprotein expressed predominantly by osteocytes that is best known to play negative role in bone formation by suppressing the Wnt signaling pathway, a canonical pathway that promotes osteogenic differentiation and osteoblast maturation and survival (62). In addition to its inhibiting effect of osteogenesis, studies also reveal its promoting effect of osteoclastogenesis and bone resorption. Wijenayaka et al. found that sclerostin could increase RANKL expression in MLO-Y4 osteocyte-like cells, while downregulating OPG, which promoted osteoclast formation and bone resorption (63). Then Ohori et al. further found that sclerostin-induced RANKL expression in primary osteocytes could be enhanced by TNF-α (40), indicating a complicated crosstalk among pro-osteoclastogenic cytokines.

#### 2.2.6 VEGF

The vascular endothelial growth factor (VEGF) family comprises five proteins: namely VEGF-A, VEGF-B, VEGF-C, VEGF-D and placental growth factor (PlGF), among which VEGF-A is the most extensively studied member. As a primary regulator of blood vessel formation and permeability, VEGF-A is involved in both physiological and pathological conditions, such as wound healing cancer (64). Recent studies reveal that VEGF also participate in bone remodeling by regulating osteoclast formation and activation. VEGF can be secreted by bone-related cells, such as osteocytes, osteoblasts, chondrocytes and macrophages, and this secretion can be enhanced by apoptotic osteocytes in the neighborhood (65). Study found that VEGF can promote osteoclast differentiation and bone resorption, which is sufficient to take the place of M-CSF (66). Yang et al. further found that VEGF could bind to VEGFR2 in OCPs and promote osteoclast formation through PI3K/Akt and MEK/ERK signaling (67). These results indicate a novel role of VEGF in osteoclast regulation.

#### 2.2.7 HMGB1

High mobility group box 1 (HMGB1) is a proinflammatory cytokine expressed and released by various cell types, including osteoclasts, osteoblasts, osteocytes, which exerts various cellular compartment‐specific functions (68). After secretion by active cells or release from damaged cells, HMGB1 serves as a protein of DAMPs and triggers a series of cellular processes, including differentiation, proliferation, apoptosis, and autophagy (68, 69). This process is mediated by its interaction with two receptors: toll‐like receptor 4 (TLR4) and receptor for advanced glycation end products (RAGE) (70). It has been reported that HMGB1 plays important role in regulating bone tissue homeostasis (13, 68, 69). Studies found that damaged MLO-Y4 osteocyte-like cells released HMGB1, which induced expression of RANKL, TNF-α, and IL-6 in other stromal cells and macrophages (65, 71). These cytokines are crucial for inducing osteoclastogenesis. HMGB1 can also directly act on OCPs and osteoclasts to induce osteoclast differentiation through RAGE and TLR4 activation (13). When activated by HMGB1, TLR4 triggers multiple signaling pathway, such as NF‐κB signaling and integrin signaling, to initiate osteoclast-related gene expression at early stage and actin ring formation at later stage (72).

#### 2.2.8 ATP

Adenosine triphosphate (ATP) is a multifunctional signaling molecule, which plays a crucial role in the metabolism of cellular energy. It is recently reported that elevated extracellular ATP engages in the development of inflammatory disease, such as osteoporosis (73). Under pathological condition such as fracture, apoptotic or damaged osteocytes release ATP, which induces bone remodeling by interacting with osteoblasts and osteoclasts (73, 74). Extracellular ATP mainly interacts with P2X purinergic receptors that are widely expressed in various kinds of bone cells. Among P2X receptors, P2X7 receptors are considered to play important role in osteoclast formation (75, 76). Activated P2X7 receptor induces the fusion of mononuclear precursors osteoclast into multinucleated osteoclast (76). Besides, P2X7 receptors activated by extracellular ATP have been found to exert effects on osteoclasts resorption by inducing the formation of resorption area on the bone surface and the secretion of osteolytic molecules, such as cathepsin K (77). These pro-osteoclastogenic effects are considered to result from the ATP-mediated rise of calcium ions in osteoclasts, which further upregulates the expression of NFATc1, the key pro-osteoclastogenic transcription factor (76). A recent study by Lu et al. revealed another mechanism that P2X7 receptors promote osteoclast formation and bone resorption via PI3K-Akt-GSK3β signaling pathway (75). While studies reported the pro-osteoclastogenic effects of ATP, some studies found the opposite results. Miyazaki et al. found that treatment of high concentration (100 μM and 150μM) of ATP analogue showed strong inhibitory effects on morphology, survival, and the bone-resorbing activity of mature osteoclasts (78). This result is consistent with a previous study revealing that extracellular ATP at low concentrations (0.2~2 μM) could promote osteoclast formation and resorption, while extracellular ATP a higher concentration (20~200 μM) might exert inhibitory effects on osteoclasts (79). These results indicate a complex role of ATP in osteoclast formation and activation. More studies are required to understand the role of extracellular ATP.

To summarize, these signal molecules are secreted by viable osteocytes, immune cells and other cells and exert regulating effects on osteoclast formation. Studies have revealed that their secretion do not cease when osteocytes die. Instead, dying or impaired osteocytes continue to regulate osteoclast formation by actively secreting or passively releasing signal molecules, which act on osteoclasts directly or indirectly. It is important to understand how osteocyte death regulates osteoclast formation.

## 3 Osteocyte death and osteoclast formation

Cell death is defined as an irreversible loss of plasma membrane integrity (80). In 2005, Nomenclature Committee on Cell Death (NCCD) classified cell death based on the morphological characteristics and specified 3 forms of cell death: apoptosis, autophagy, and necrosis (81). As more and more forms of cell death have been identified, previous classification become inapplicable. Currently, cell death is classified into two main forms: non-programmed cell death (i.e. necrosis) and programmed cell death (PCD), which is further divided into apoptosis, pyroptosis, necroptosis and ferroptosis, etc., based on different pathological process (82). Unlike accidental and unregulated process of necrosis, PCD is a spontaneous and programmed process of cell death under the regulation of certain gene and protein, which can be blocked by the corresponding inhibitors (83). Studies have revealed the crucial role of cell death in the development of diseases (84, 85). Various forms of osteocyte death, including apoptosis, necroptosis, ferroptosis and pyroptosis, have been reported to participate in multiple bone diseases, especially inflammation and bone loss, where osteoclasts are excessively generated and activated (86). As previous studies have reported that death of osteocytes recruit osteoclasts and promote bone resorption (87), it is important to sort out the mechanisms of various osteocyte death to regulate osteoclast. Some mechanisms have been verified by current studies, while some remain unclear. These mechanisms, established or potential, are discussed as following (**Table 2**).

**Table 2:** Comparison of various types of osteocyte death.

Osteocyte death	Triggering factors	Key pathway	Outcome	Released pro-osteoclastogenic factors
Necrosis	Mechanical force, heat, cold, ischemia, etc.	No	Cell membrane rupture; Release of cellular contents; Triggering inflammation	HMGB1 ^pt,d,id^, S100 family molecules ^id^, heat-shock proteins ^id^
Apoptosis	Aging, excess glucocorticoids, estrogens deficiency, inflammation, fatigue/microdamage, unloading/disuse, death receptors ligands, etc.	Caspase activation cascade	Secondary necrosis	RANKL ^d^, TNF-α ^d,id^, IL-6 ^id^, VEGF-A ^d^, ICAM-1 ^d^, HMGB1 ^d,id^, ATP ^id^
Necroptosis	Death receptors ligands, etc.	RIPK1/RIPK3/MLKL	Cell membrane rupture; Release of cellular contents; Triggering inflammation	DAMPs (HMGB1 ^pt^) ^d,id^
Ferroptosis	Iron overload, etc.	Fe^2+^/ROS	Peroxidation of lipid, DNA, proteins; Cell membrane rupture ^pt^	RANKL ^d^, M-CSF ^d^, IL-1β ^d,id^, IL-6 ^d,id^, TNF-α ^d,id^, DAMPs (HMGB1 ^pt^) ^d,id^
Pyroptosis	PAMPs, DAMPs, LPS, etc.	Caspase/GSDMD	Cell membrane rupture; Release of cellular contents; Triggering inflammation	IL-1β ^id^, IL-18 ^id^, DAMPs (HMGB1 ^pt^) ^d,id^

HMGB1, high mobility group box 1; RANKL, receptor activator of nuclear factor-kappa B ligand; TNF-α, tumor necrosis factor alpha; IL, interleukin; VEGF, vascular endothelial growth factor; ICAM-1, intercellular cell adhesion molecule-1; ATP, adenosine triphosphate; RIPK, receptor-interacting serine/threonine protein kinase; MLKL, mixed lineage kinase domain-like; DAMPs, damage associated molecule patterns; ROS, reactive oxygen species; M-CSF, macrophage colony stimulating factor; PAMPs, pathogen-associated molecular patterns; LPS, lipopolysaccharides; GSDMD, gasdermin D; ^d^ directly promoting osteoclastogenesis; ^id^ indirectly promoting osteoclastogenesis; ^pt^ Potentially.

### 3.1 Necrosis

Necrosis is a uncontrolled and pathological form of cell death that is caused by supraphysiological conditions, such as mechanical force, heat, or cold, and morphologically characterized by plasma membrane rupture and dilatation of cytoplasmic organelles, particularly mitochondria (88). Rupture of cell membrane leads to a release of intracellular contents (i.e. DAMPs) and induces aseptic inflammatory responses (20–23).

Osteocyte necrosis participates in the regulation of osteoclast formation and development of multiple bone diseases. Andreev et al. found that osteocyte necrosis, rather than apoptosis, significantly increased osteoclastogenesis and promoted bone loss (89). They further found that osteoclasts sensed DAMPs from necrotic osteocytes by macrophage-inducible C-type lectin (Mincle), a member of pattern recognition receptors (PRRs) that were subsequently found to upregulate the expression of gene related to oxidative phosphorylation and calcium signaling in osteoclast. However, they did not identify the specific molecules of DAMPs in the regulation of osteoclastogenesis. Gkouveris et al. reported that osteocyte death led to release of HMGB1 in mice model of medication-related osteonecrosis of the jaw (MRONJ) and blocking HMGB1/RAGE interaction would reduce the incidence of MRONJ (90). Therefore, necrotic osteocytes might regulate osteoclastogenesis via the interaction between DAMPs released from dead osteocytes, such as HMGB1, and PRRs on osteoclasts, such as MINCLE. It is also reported that DAMPs released from necrotic osteocytes, such as HMGB1, S100 family molecules, and heat-shock proteins, could bind to PRRs, such as TLR2, TLR4, and RAGE, on immune cells including macrophages and dendritic cells, and promote their production of pro-inflammatory cytokines, such as TNF-α and IL-6, which further induces RANKL expression in osteoblast lineage cells or promotes osteoclastogenesis directly (91). Meanwhile, necrotic osteocytes might indirectly promote osteoclastogenesis by exerting pro-neovascularization effect. Osteonecrosis of the femoral head (ONFH) is a bone disease with a progressive pathological process of femoral head ischemia, involving not only osteocyte necrosis, but hyperactive osteoclasts (92, 93). Varoga et al. found VEGF expression in osteocytes was upregulated and immature vessels increased in penumbra area of ONFH, where osteocytes are still alive (94). This result suggests that necrotic osteocytes might promote VEGF expression in nearby surviving osteocytes and thereby trigger neovascularization, which might promote signal transduction and recruit osteoclasts. Meanwhile, given that VEGF is currently found to participate in the regulation of osteoclast formation as discussed previous, it is potential that necrotic osteocytes regulate osteoclast formation via the direct effects of VEGF on osteoclasts. However, a recent study found that sclerostin was significantly upregulated while VEGF decreased in necrotic area of the femoral head induced by glucocorticoid, and silencing sclerostin of osteocytes rescued the expression of osteogenic and angiogenic gene (95). Thus, it requires further studies to verify the effects of necrotic osteocytes on angiogenic gene expression. To summarize, necrotic osteocytes can promote osteoclast formation by releasing DAMPs which directly stimulate osteoclasts or trigger the expression of pro-osteoclastogenic cytokines in related cells.

### 3.2 Apoptosis

**Figure 2:**
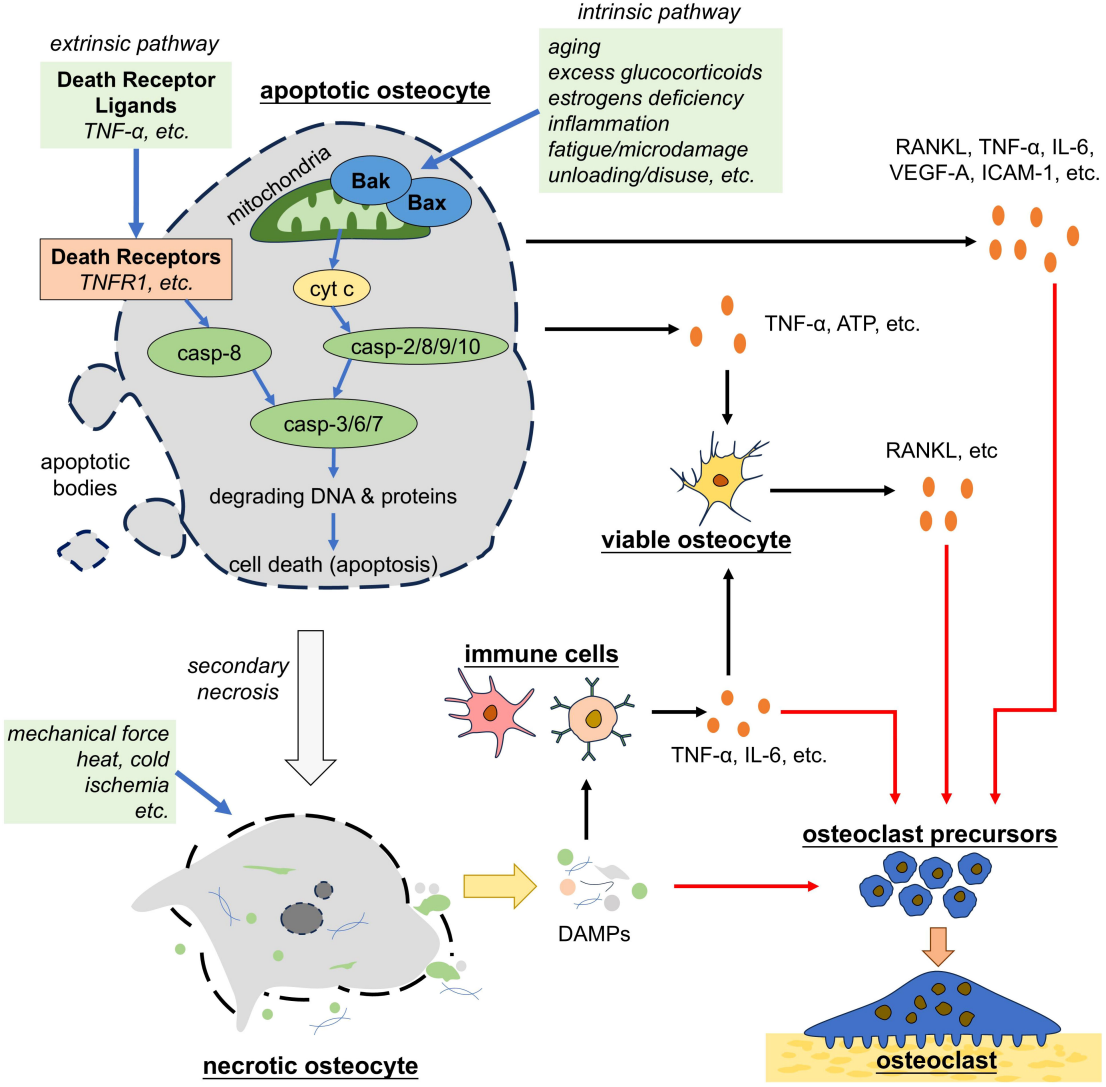
Apoptotic and necrotic osteocytes promote osteoclastogenesis. Osteocytes that undergo apoptosis by either the intrinsic or extrinsic pathways trigger the secretion of pro-osteoclastogenic factors by nearby osteocytes or directly promote osteoclastogenesis. Apoptotic osteocytes eventually undergo secondary necrosis. Necrotic osteocytes, either derived from apoptotic osteocytes or induced by environmental disadvantages, lose the integrity of cell membrane and release the cellular contents (i.e. DAMPs), which trigger inflammation and osteoclastogenesis. TNF-α, tumor necrosis factor alpha; casp, caspase; ATP, adenosine triphosphate; RANKL, receptor activator of nuclear factor-kappa B ligand; IL-6, interleukin 6; VEGF, vascular endothelial growth factor; ICAM-1, intercellular cell adhesion molecule-1; DAMPs, damage associated molecule patterns.

In contrast to necrosis, apoptosis is a form of programmed cell death that is best studied as a vital physiological process critical for maintaining cellular homeostasis across tissues, characterized by cell shrinkage, chromatin condensation, and membrane blebbing (96). Apoptosis is a caspase-dependent form of programmed cell death that is activated by two main pathways: the intrinsic and extrinsic pathways (**Figure 2**) (65). Intrinsic pathway is triggered by pathological conditions, such as DNA damage, hypoxia and loss of integrin signaling and begins with the activation of Bax and Bak, the pro-apoptotic members of the Bcl-2 family of proteins. Activation of Bax and Bak leads to a conformational change that exposes cryptic epitopes at their N-termini, which promotes the formation of Bax and Bak homo-oligomers or hetero-oligomers within the outer mitochondrial membrane (96, 97). These oligomers form channels that release cytochrome c from the mitochondria into the cytoplasm. Then cytochrome c activates latent caspases, a family of endopeptidases, among which the initiator caspases (caspase-2/8/9/10) are primarily activated and the effector caspases (caspase-3/6/7) are subsequently activated. Effector caspases are responsible for demolition of the cell. Caspase-3/6/7 degrade critical intracellular proteins, and activate other enzymes including DNase (98, 99). Extrinsic pathway of apoptosis is triggered by the interaction between death receptors ligands (such as TNF-α), and death receptors (such as tumor necrosis factor receptor 1, TNFR1), which recruit and activate caspase-8, the key protein for the following caspase activation cascade as introduced above (100). Apoptotic cells eventually break apart into vesicles, which are called apoptotic bodies and are usually phagocytosed by macrophages or other cell types. Phagocytose requires the guidance of “find-me” signals and “eat-me” signals. Apoptotic cells actively release soluble molecules, such as Sphingosine-1-phosphate (S1P), ATP, uridine triphosphate (UTP), Lysophosphatidylcholine (LPC) and C-X3-C motif chemokine ligand 1 (CX3CL1). These molecules attract macrophages to the site of cell death, thus known as “find-me” signals (101). Macrophages in proximity to apoptotic cells recognize “eat-me” signals displayed on the surface of apoptotic cells, such as Fc and phosphatidyl serine (PS) (101). During apoptosis, the PS molecules within the phospholipid bilayer of cell surface membrane undergo a translocation from inner to outer surface of the bilayer, which are recognized by macrophages and then facilitate phagocytosis (65). Following this regulated process, apoptosis ensures the removal of damaged or unnecessary cells without inciting inflammatory responses (96).

Osteocyte apoptosis is recognized as a pivotal event in bone remodeling. Recent research has highlighted that osteocyte apoptosis is triggered by pathological conditions such as aging, excess glucocorticoids, estrogens deficiency, inflammation, fatigue/microdamage, and unloading/disuse (65). Aging increased oxidative stress and mitochondrial dysfunction, driving osteocyte apoptosis and subsequently diminishing the structural integrity of bone (102). Excessive glucocorticoid use and estrogen deficiency are reported to induce osteocyte apoptosis and cause bone loss (103, 104). Under inflammatory condition, inflammatory cytokines, particularly TNF-α, increase osteocyte apoptosis and lead to osteoporosis, a disease characterized by elevated bone fragility (105). In addition to systemic factors, local mechanical unloading or disuse significantly promotes osteocyte apoptosis (106).

Normally, apoptotic bodies are engulfed by macrophages timely and therefore not inducing wide range of inflammatory response, but osteocytes are located within bony lacunae, making apoptotic osteocytes unapproachable for macrophages (89). Under physiologic condition, osteocytes connect to bone surface and each other via the canalicular network. However, study found that few apoptotic bodies could traverse this canalicular network occupied by the dendritic processes of surrounding viable osteocytes, although it is theoretically accessible (107). As many studies have indicated that apoptotic osteocytes promote osteoclast mediated bone resorption (10, 108), there must be non-contacting approach by which apoptotic osteocytes recruit and activate osteoclast, since their apoptotic bodies are unapproachable for macrophages, at least at the early stage.

Current studies have revealed that apoptotic osteocytes regulate osteoclast formation in both active and passive way. Osteocytes undergoing apoptosis increase the secretion of the pro-osteoclastogenic factors, including RANKL, TNF-α, IL-6, VEGF-A, intercellular cell adhesion molecule-1 (ICAM-1) (13, 14, 55, 109, 110). These factors exert dual effects on osteoclasts directly or indirectly. As discussed in previous passage, RANKL, TNF-α, IL-6, VEGF-A act on OCPs and promote their differentiation to mature osteoclasts as well as the subsequent activation of bone resorption. IL-6 and ICAM-1 are reported to promote the adhesion of OCPs to endothelial cells (55). Besides, apoptotic osteocytes can signal to nearby osteocytes and macrophages to stimulate their secretion of pro-osteoclastogenic factors, such as RANKL, TNF-α, IL-1β, IL-6, IL-8, and VEGF (65, 111). It is reported that dead osteocytes accounted for only about 15~20 percent even in the regions showing the highest apoptosis induced by estrogen loss (112). Verborgt et al. found that osteocytes surrounding the apoptotic osteocyte at microdamage sites upregulated the expression of apoptosis-inhibiting gene (113), showing that surviving osteocytes could detect apoptotic osteocytes and potentially continue to exert regulating effects on bone remodeling. TNF-α secreted by apoptotic osteocytes acts on TNFRs in viable osteocytes in the neighborhood, which further activates mitogen-activated protein kinases (MAPKs) phosphorylation and the NF-κB pathway to increase expression of RANKL and sclerostin, both of which subsequently initiate osteoclast formation (39, 40). Additionally, studies found that apoptotic osteocytes induced by fatigue loading released ATP, which triggered nearby osteocytes to express RANKL and induced osteoclast-mediated bone resorption (114, 115). To summarize, apoptotic osteocytes actively regulate osteoclast mediated bone resorption in both direct and indirect way. However, it requires more studies to reveal the mechanism by which these signal molecules are upregulated in apoptotic osteocytes and further explore the strategy to block the secretion of pro-osteoclastogenic factors by apoptotic osteocytes.

Apoptotic osteocytes also regulate osteoclast-mediated bone resorption in a passive way. Free from phagocytosis by macrophages, apoptotic osteocytes undergo secondary necrosis, leading to the release of DAMPs, including HMGB1, which further trigger the release of pro-osteoclastogenic cytokines in osteoblast and stromal cells (116). As discussed previously, HMGB1 stimulates the synthesis of RANKL, TNF-α, and IL-6, but inhibits the production of OPG in bone marrow-derived stromal cells (65, 69, 71). Besides, HMGB1 can also directly act on TLR4 in OCPs and osteoclasts to trigger osteoclast-related gene expression at early differentiation stage and actin ring formation at later stage (13, 72).

In conclusion, osteocyte apoptosis plays important role in osteoclast regulation. However, Plotkin et al. found that inhibition of osteocyte apoptosis was not sufficient to prevent the osteoclast increase or bone mass decrease induced by unloading (117), indicating another advantaged mechanism of regulating osteoclasts. He et al. found that compared with apoptosis, necroptosis, a novel form of programmed cell death, took more responsibility for osteocyte death in ovariectomy (OVX)-induced osteoporosis (118). This may account for the insufficiency of inhibiting osteocyte apoptosis to prevent osteoclast increase. Thus, focus should be put not only on osteocyte apoptosis, but also on other types of programmed osteocyte death.

### 3.3 Necroptosis

**Figure 3:**
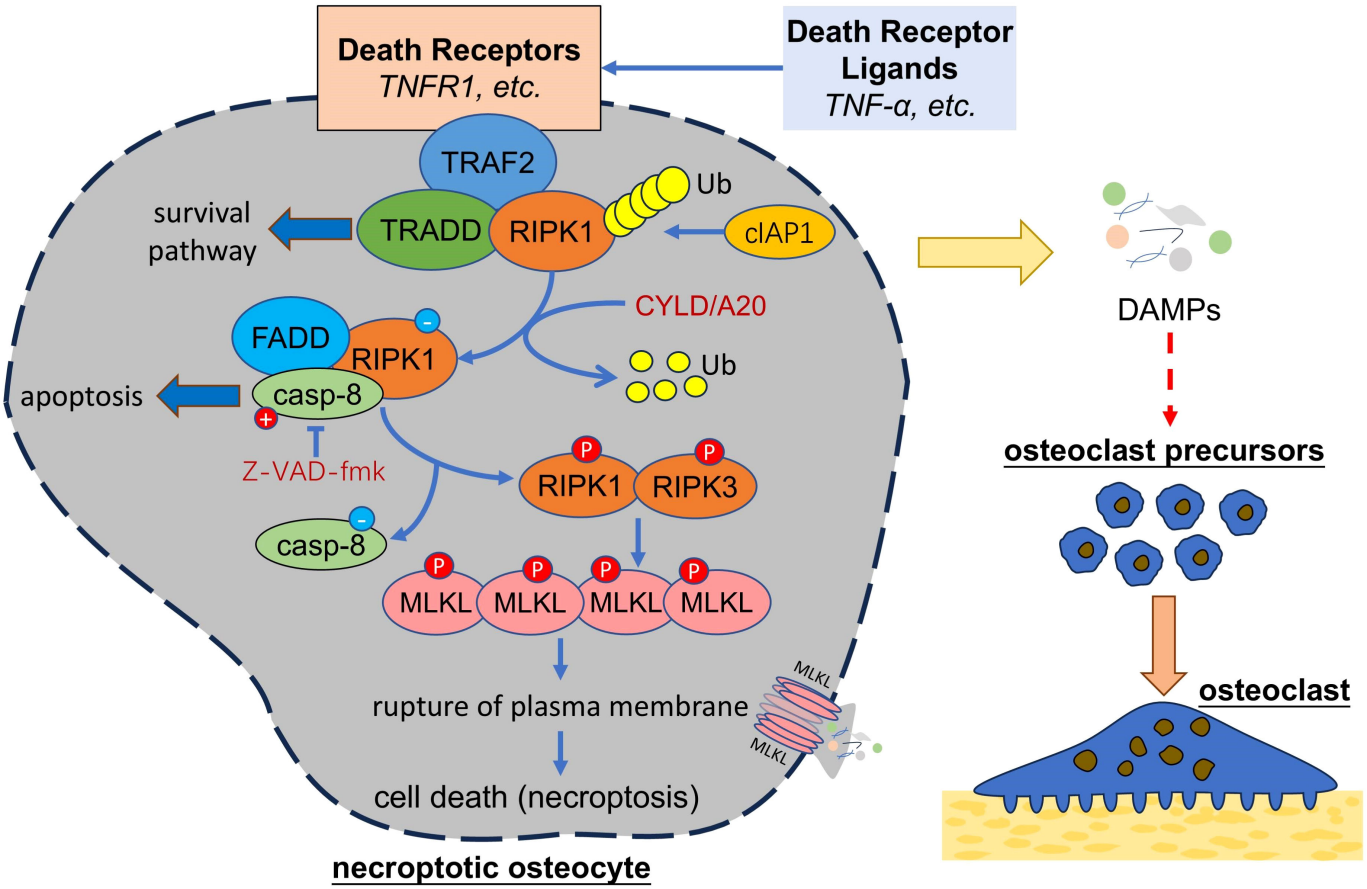
Necroptotic osteocytes promote osteoclastogenesis. Necroptosis of osteocytes can be triggered by death receptor ligands, which activate a series of signal transduction and activate RIPK1 and RIPK3 under certain condition. RIPK1-RIPK3 interaction can activate MLKL, which oligomerize and damage the integrity of plasma membrane, leading to cell death and release of cellular contents (i.e. DAMPs) that might further trigger the osteoclastogenesis. TNF, tumor necrosis factor; TRAF2, TNFR-associated factor 2; RIPK, receptor-interacting serine/threonine protein kinase, TRADD, TNFR-associated death domain; cIAP1, cellular inhibitor of apoptosis protein 1; CYLD, Cylindromatosis; FADD, Fas associated via death domain; casp-8, caspase-8; MLKL, mixed lineage kinase domain-like; DAMPs, damage associated molecule patterns.

It was thought that apoptosis was the only form of programmed cell death. In 2005, Degterev et al. identified a form of cell death which was characterized by necrotic cell death morphology but could be inhibited by necrostatin-1(Nec-1). They termed it as “necroptosis” and classified it as a novel form of programmed cell death which is distinct from apoptosis (119). Necroptosis proceeds in a programmed way, rather than in an unregulated way as happened in necrosis, and undergoing in a Caspase-independent manner, rather than in a Caspase-dependent manner as happened in apoptosis (**Figure 3**). Necroptosis can be trigger by TNF family, Fas ligands, interferon, and RNA or DNA sensors (120). TNF-α and FAS, two death receptor ligands, are the best studied inducers of necroptosis. Activated by TNF-α, TNFR1 on the cell membrane recruits receptor-interacting serine/threonine protein kinase 1 (RIPK1), TNFR-associated death domain (TRADD), TNFR-associated factor 2 (TRAF2) and form complex I (120), which is involved in the survival pathway and contributes to cell survival. This process is dependent on the ubiquitination of RIPK1 by cellular inhibitor of apoptosis protein 1 (cIAP1), so when RIPK1 is deubiquitinated by Cylindromatosis (CYLD) or deubiquitination enzyme A20, complex I will be converted into complex Il, which is composed of Fas associated via death domain (FADD), RIPK1 and caspase-8 (121). Activated caspase-8 in complex Il degrades RIPK1 and initiates apoptosis, but when caspase-8 is inhibited (eg, inhibited by Z-VAD-fmk), RIPK1 recruits RIPK3 and the interaction between RIPK1 and RIPK3 further recruits and phosphorylates mixed lineage kinase domain-like (MLKL) (122). Phosphorylated MLKL oligomerizes and translocates into the plasma membrane, leading to the rupture of plasma membrane and cell death (123). As happened in necrosis, cell necroptosis ultimately causes a release of DAMPs and induces inflammatory responses.

Osteocyte necroptosis is recently found involved in the development of bone diseases like osteoporosis (124). It is reported that both necroptosis and apoptosis were found responsible for death of osteocytes and bone loss in OVX mice, the model of postmenopausal osteoporosis, but necroptosis might hold more responsible on the death of osteocytes than apoptosis (118). As osteoclasts were found increased in OVX mice (125), it might be a relation of osteocyte death, especially necroptosis, to osteoclastogenesis. Although a few studies have revealed how osteocyte necroptosis triggers osteoclastogenesis, given that necroptosis of osteocytes ultimately leads to the rupture of cell membrane and release of DAMPs, it is potential that DAMPs released from necroptotic osteocytes recruit OCPs and trigger osteoclastogenesis, as happened in necrosis or apoptosis of osteocytes. HMGB1 has been identified as one of the DAMPs released from necroptotic cells (126). As discussed previously, impaired osteocytes release HMGB1 and trigger osteoclastogenesis. It is potential that necroptotic osteocytes release HMGB1 as the major regulator of osteoclastogenesis. Besides, Zhu et al. found that knockdown of RIPK1, one of the key regulators of necroptosis, down-regulated VEGF-D gene and protein levels in astrocytes (127). Similar to necrosis, it is potential that necroptotic osteocytes regulate osteoclast formation via VEGF related signaling pathway. To summarize, necroptotic osteocytes might promote osteoclast formation by releasing DAMPs or upregulating the expression of pro-osteoclastogenic cytokines. However, it should be reminded that in necroptosis-inducing environment, high level of TNF-α might induce RANKL production in osteocytes ahead of the complete death, which would trigger osteoclastogenesis (39). Thus, studies are required to distinguish between osteoclastogenesis triggered by TNF-α-induced RANKL secretion and by osteocyte necroptosis-induced DAMPs release.

Though it remains uncertain how necroptotic osteocytes induce osteoclast formation, studies have revealed the potential target of osteocyte necroptosis as clinical application for relevant bone diseases. Cui et al. found the treatment of Nec-1, an inhibitor of RIPK1, alleviated bone loss in OVX mice, indicating the potential application of Nec-1 in the treatment of postmenopausal osteoporosis (128). Similar result was found in the rat of glucocorticoid induced osteoporosis (129). In addition to RIPK1 inhibitors, there are other inhibitors of necroptosis, such as RIPK3 inhibitor GSK’872 and MLKL inhibitor Necrosulfonamide (124). However, their efficacy in the treatment of relevant bone diseases and safety for human being requires further investigation.

### 3.4 Ferroptosis

**Figure 4:**
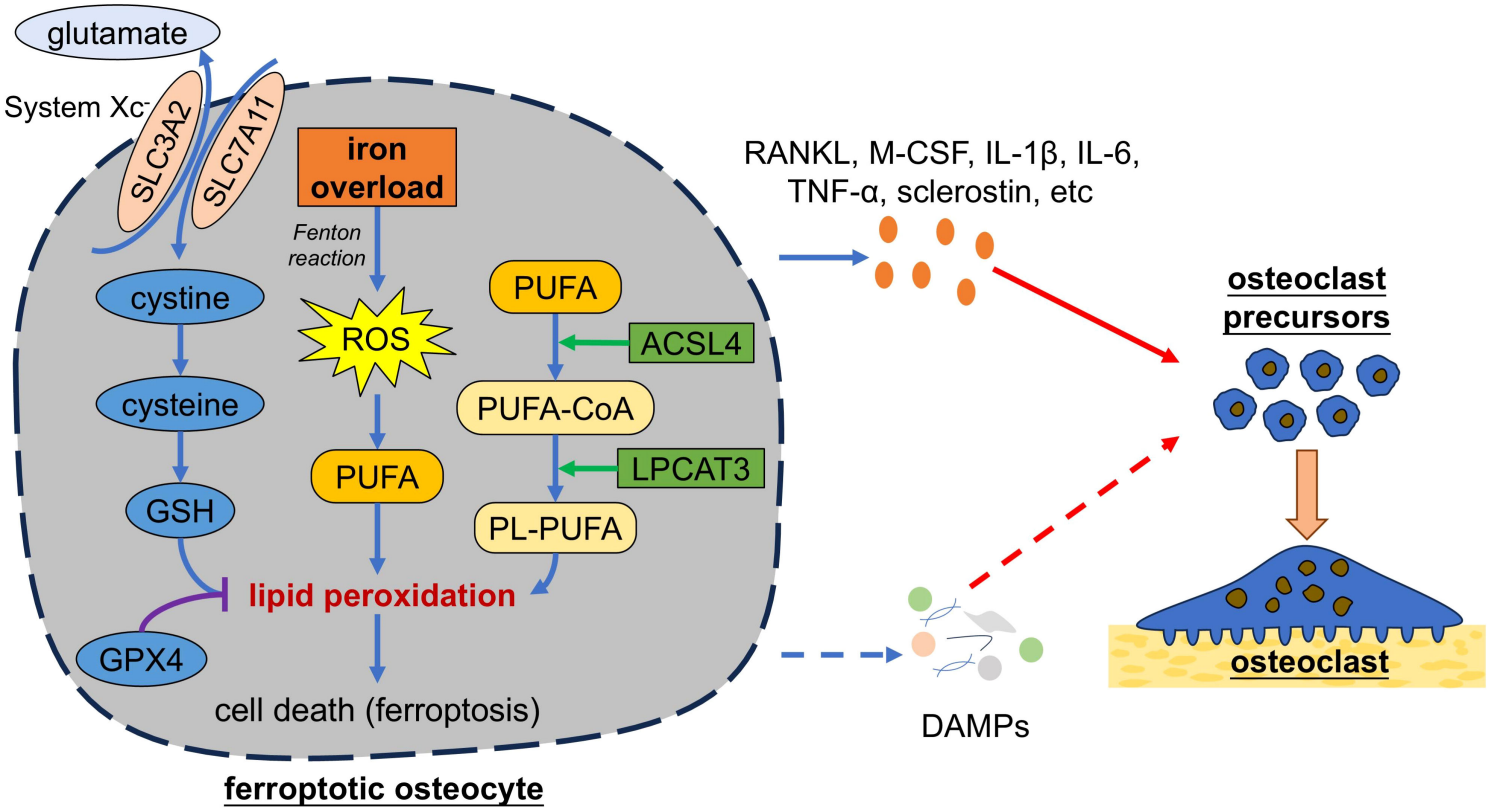
Ferroptotic osteocytes promote osteoclastogenesis. Iron overload causes excessive generation of ROS in osteocytes. Excessive ROS can cause oxidative damage to membrane lipids and ultimately lead to cell death. Dysregulation of lipid metabolism mediated by ACSL4 and LPCAT3 can also cause ferroptosis. Ferroptosis can be regulated by several signaling pathway, such as system Xc^-^/GSH/GPX4 axis. Ferroptotic osteocytes secrete pro-osteoclastogenic factors to trigger osteoclastogenesis. Additionally, ferroptotic osteocytes might release cellular contents (i.e. DAMPs) and thereby trigger the osteoclastogenesis. SLC3A2, solute carrier family 3 members 2; ROS, reactive oxygen species; GSH, glutathione; GPX4, glutathione peroxidase 4; PUFAs, polyunsaturated fatty acids; ACSL4, acyl-CoA synthetase long-chain family member 4; LPCAT3, lysophosphatidylcholine acyltransferase 3; PL, phospholipid; RANKL, receptor activator of nuclear factor-kappa B ligand; M-CSF, macrophage colony stimulating factor; TNF-α, tumor necrosis factor alpha; IL, interleukin; DAMPs, damage associated molecule patterns.

In addition to apoptosis and necroptosis, more forms of programmed cell death are identified. In 2003, Dolma et al. found that the small molecule erastin can induce a novel form of cell death, which was further found unable to be reversed by neither apoptosis inhibitors nor necroptosis inhibitors, while iron-chelating agent deferoxamine mesylate (DFO) could inhibits such form of cell death. Thus, a new form of iron-dependent cell death was identified and named as ferroptosis (130). Different from apoptosis and necroptosis, ferroptosis is characterized by the increase of the mitochondrial membrane density with mitochondrial shrinkage and triggered by the dysregulation of iron homeostasis and lipid metabolism (**Figure 4**) (131, 132). Normally, extracellular Fe^3+^ binds to transferrin (TF), which can transport Fe^3+^ into cells through TF receptor 1 (TFR1). Intracellular Fe^3+^ is then reduced to Fe^2+^ by iron reductase, such as six-transmembrane epithelial antigens of the prostate 3 (STEAP3) and divalent metal transporter 1 (DMT1) (133). Fe^2+^ is unstable and able to generates high level of reactive oxygen species (ROS) via the Fenton reaction (134). Iron overload leads to excessive ROS, which, if the antioxidant capacity of cells is insufficient, further causes oxidative damage to polyunsaturated fatty acids (PUFAs) in cell membrane, DNA, proteins, and ultimately cell death (i.e. ferroptosis) (134, 135). Dysregulation of lipid metabolism can also cause ferroptosis, where acyl-CoA synthetase long-chain family member 4 (ACSL4) and lysophosphatidylcholine acyltransferase 3 (LPCAT3) play important roles (136). ACSL4 promotes the conversion of free PUFAs to PUFA-CoAs and then LPCAT3 catalyze the formation of phospholipid-PUFAs (PL-PUFAs) from PUFA-CoAs. PL-PUFAs are highly prone to be oxidized into lipid peroxides, thereby making cells susceptible to ferroptosis. Ferroptosis is regulated by several signaling pathway, including system Xc^-^/glutathione (GSH)/glutathione peroxidase 4 (GPX4) axis (137). System Xc^-^ is located on the cell membrane and composed of the transporter protein solute carrier family 7 members 11 (SLC7A11) and regulator protein SLC3A2. System Xc^-^ is responsible for exporting intracellular glutamate in exchange for extracellular cystine, which is further reduced to cysteine for the synthesis of GSH, the main antioxidant in cells (138). GSH is an essential cofactor for GPX4, which reduces the toxicity of lipid peroxides by converting peroxide (R-OOH) into alcohol (R-OH), thereby protecting cells from ferroptosis (132). Erastin can inhibit system X_c_^-^ and induce ferroptosis (139).

Increasing studies have revealed the important role of iron overload and ferroptosis in bone diseases like postmenopausal osteoporosis, where increased oxidative stress and iron accumulation is found (16). While previous studies focused on ferroptosis of osteoblasts in bone diseases, a recently increasing number of studies have paid attention on osteocytes. Yang et al. reported that iron overload induced by ferric ammonium citrate (FAC) could lead to osteocyte apoptosis and conditioned medium from iron-overload-induced osteocytes could promote osteoclast differentiation and activity of bone resorption. They further revealed that iron overload significantly increased the gene expression and protein secretion of RANKL in osteocytes, which promoted osteoclast differentiation and activation (140). Similar results were found by Ma et al. and Guo et al (141, 142). Ma et al. found that osteocyte apoptosis induced by iron overload led to an increasing expression of RANKL and sclerostin with a decreased expression of OPG both *in vivo* and *in vitro*, while Guo et al. reported that iron overload upregulated RANKL expression with no significant impact on OPG and sclerostin levels *in vivo*. These results suggest that iron overload could induce osteocyte death and promote osteoclast differentiation and activation by increasing RANKL expression. However, as increasing studies have revealed the relation between iron overload and ferroptosis (137), these studies that focused on osteocyte apoptosis might neglect the significance of osteocyte ferroptosis in osteoclast regulation. A recent study by Jiang et al. reported that iron overload induced osteocyte death in OVX mice, which could be decreased by ferroptosis inhibitor, but not by apoptosis inhibitor, neither by necroptosis inhibitor, therefore confirming that ferroptosis, instead of apoptosis or necroptosis, was the predominant form of death among osteocytes in a high-iron environment (16). They also found that osteocytes induced by iron overload significantly increased the secretion of RANKL, M-CSF, IL-1β, IL-6, and TNF-α, which could be inhibited by ferroptosis inhibitor liproxstatin-1 (Lip-1) and ferrostatin-1 (Fer-1), indicating that osteocyte ferroptosis could promote osteoclast formation by secreting pro-osteoclastogenic cytokines. Consistently, they found that iron overload could lead to bone loss, which could be suppressed by ferroptosis inhibitor. Their further study revealed the crucial role of nuclear factor erythroid derived 2-related factor-2 (Nrf2) in the regulation of osteocyte-ferroptosis-induced osteoclastogenesis, as inhibition of Nrf2 downregulated DNA methyltransferase 3a (Dnmt3a)-mediated the methylation levels of RANKL promoter and thereby reduced the expression of RANKL. These results provide a novel insight for therapy of postmenopausal osteoporosis by targeting osteocyte ferroptosis. Similar results were found in the studies of periodontitis. Li et al. identified the occurrence of alveolar osteocyte ferroptosis in diabetic periodontitis mice (143). Their further *in vitro* study revealed the increased protein expression of TNF-α and IL-1β in osteocytes, as well as the upregulated mRNA expression of proinflammatory cytokines, including sclerostin, IL-6, TNF-α and RANKL, which could be reversed by ferroptosis inhibitor. These results are consistent with study by Tang et al (144).

There are some studies implying the potential mechanism by which ferroptosis osteocytes regulate osteoclasts. Li et al. found triggering the ferroptosis of pancreatic ductal adenocarcinoma cells would induce the release of HMGB1 (145), which facilitated the pro-inflammatory M1 polarization of macrophages via HMGB1/TLR4/STAT3 axis (146). Wei et al. found the similar result that ferroptosis of acute myeloid leukemia cells could lead to the release of HMGB1 (147). Additionally, it is reported that cancer cells undergoing ferroptosis released proteoglycan decorin, which triggered the production of pro-inflammatory cytokines in macrophages via NFKB/NF-κB axis (148). These results indicate that ferroptosis osteocytes might also release DAMPs that participate in osteoclast regulation, which requires further studies to explore.

To summarize, iron overload-induced osteocyte ferroptosis plays important role in the regulation of osteoclast formation and bone resorption activity. However, it should be taken into consideration that high-iron environment could act on osteoclasts directly. Osteoclast differentiation and activation require energy provided by mitochondria, while mitochondrial biogenesis requires iron. Thus, high level of iron might promote osteoclast differentiation and activation, thereby contributing to bone resorption (149). Das et al. found that suppressing iron uptake of osteoclast attenuated their mitochondrial metabolism and cytoskeletal organization, leading to decreased bone resorption (150). This result indicates the important of role of iron in the regulation of osteoclast-mediated bone resorption and is consistent with the study by Li et al. reporting that isobavachin, a RANKL inhibitor, could suppress RANKL-induced osteoclastogenesis by promoting iron efflux and inhibiting mitochondrial biogenesis (151). Therefore, a comparison between the effect of osteocyte ferroptosis and iron overload on regulating osteoclast differentiation and bone resorption is required to clarify the exact role of osteocyte ferroptosis in osteoclast regulation, which would help to provide an osteocyte ferroptosis-targeting insight for therapy of osteoclast-mediated bone diseases.

### 3.5 Pyroptosis

**Figure 5:**
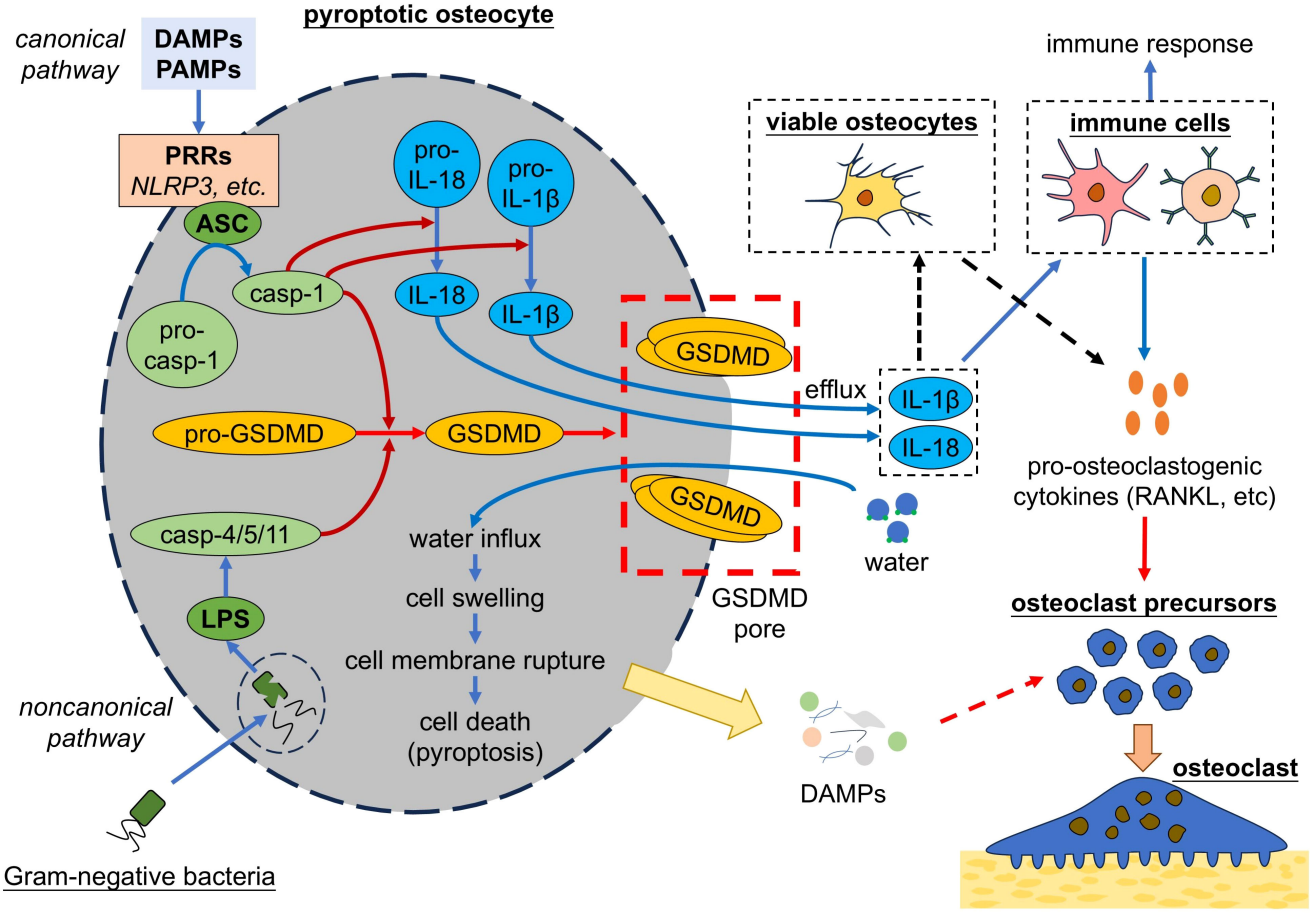
Pyroptotic osteocytes promote osteoclastogenesis. Osteocytes undergo pyroptosis by either canonical pathway or noncanonical pathway, both of which lead to the activation of GSDMD. GSDMD form pores in cell membrane, leading to cell membrane rupture and cell death. Pyroptotic osteocytes release pro-inflammatory cytokines, which act on immune cells to trigger the immune response and secretion of pro-osteoclastogenic cytokines. Additionally, pyroptotic osteocytes might release cellular contents (i.e. DAMPs) and thereby trigger the osteoclastogenesis. DAMPs, damage associated molecule patterns; PAMPs, pathogen-associated molecular patterns; PRRs, pattern recognition receptors; NLRP3, nucleotide-binding oligomerization domain-like receptor family pyrin domain containing 3; ASC, apoptosis-associated speck-like protein containing a CARD; casp, caspase; IL, interleukin; LPS, lipopolysaccharides; GSDMD, gasdermin D; RANKL, receptor activator of nuclear factor-kappa B ligand.

Pyroptosis is another form of programmed cell death, which is triggered by infections or inflammatory signals and results in the rupture of the cell membrane. Two pyroptosis pathway have been identified: Canonical pathway and noncanonical pathway (**Figure 5**). Canonical pyroptosis is triggered by the interaction between PRRs and pathogen-associated molecular patterns (PAMPs) or DAMPs, which induces the formation of inflammasome that consists of nucleotide-binding oligomerization domain-like receptor family pyrin domain containing 3 (NLRP3), apoptosis-associated speck-like protein containing a CARD (ASC) and pro-caspase-1 (152). Inflammasome activates caspase-1, which cleaves gasdermin D (GSDMD), pro-IL-1β and pro-IL-18 into their mature forms. Then GSDMD form pores in cell membrane that allow water influx and induce cells to swell until the cell membrane ruptures, ultimately leading to the release of cellular contents and triggering strong inflammatory response (153). GSDMD-pores also allow efflux of pro-inflammatory factors, such as IL-1β and IL-18 (154). Noncanonical pyroptosis is triggered by lipopolysaccharides (LPS) of Gram-negative bacteria and characterized by the activation of caspase-4/5/11, which activate GSDMD and lead to cell death as happened in canonical pyroptosis (155). Cell pyroptosis induces strong immune reactions in response to host defenses infection, but excessive pyroptosis can lead to multiple inflammatory diseases, including bone diseases like osteoporosis (156).

A few studies have focused on relation of osteocyte pyroptosis to osteoclast regulation. Zhao et al. found that osteocyte pyroptosis promoted osteoclast formation and bone resorption (157). Although their study did not further explore the mechanism, they found that osteocyte pyroptosis led to upregulated secretion of IL-1β, an inflammatory cytokine that can increase RANKL expression and thereby promote osteoclastogenesis (156). Similarly, Zhang et al. reported pyroptosis of MLO-Y4 osteocyte-like cells led to increased release of lactate dehydrogenase (LDH), IL-1β and IL-18 (158). IL-18 is reported to upregulate RANKL in T lymphocytes, indirectly stimulating osteoclast formation (159). These results suggest that osteocyte pyroptosis can promote osteoclastogenesis and bone resorption, probably by release of pro-inflammatory cytokines including IL-1β and IL-18, which induced upregulation of RANKL expression. There are multiple studies on how pyroptosis of various cells regulate osteoclasts, which may help to understand the relation of osteocyte pyroptosis and osteoclast regulation. It is reported that osteoblast pyroptosis induced by IL-17 promoted the release of IL-1β and RANKL, which further promoted osteoclastogenesis and bone resorption (160). Similarly, pyroptosis of periodontal ligament cells was found to promote osteoclast-mediated bone resorption, probably by IL-1β-induced upregulation of RANKL (161, 162). These results indicate the important role of pro-inflammatory cytokines including IL-1β and IL-18 in osteoclast regulation, while there might be other potential mechanism. As presented above, pyroptosis of MLO-Y4 osteocyte-like cells released LDH (158). However, such a large molecule is unlikely to pass through GSDMD pores formed during the process of pyroptosis, but possibly through cell membrane rupture sites at the later stage of pyroptosis (163), suggesting that pyroptotic cells may undergo cell membrane rupture and release DAMPs. Li et al. reported that pyroptotic renal tubular cells released HMGB1 and then triggered inflammatory responses (164). It is possible that pyroptotic osteocytes promote osteoclast formation and bone resorption by releasing DAMPs, such as HMGB1, which need more studies to explore.

## 4 Conclusion and prospects

Viable osteocytes serve as a crucial regulator of bone remodeling by translating environmental alterations into cellular signals which are further transmitted to related cells including osteoblasts and osteoclasts to initiate their activity of bone formation and resorption, respectively. This regulatory effect of osteocytes does not vanish when osteocytes die; instead, dying or dead osteocytes continue to regulate osteoclast formation in various ways and thereby affect bone remodeling. As multiple forms of osteocyte death have been identified in various bone diseases, their roles on osteoclast regulation are also revealed by current research. This review summarizes the established and potential mechanisms by which osteocyte death, including necrosis, apoptosis, necroptosis, ferroptosis and pyroptosis, regulate osteoclast formation, which might provide better understanding of pathological bone remodeling and the development of bone diseases. Whereas, there are still some questions needed to be answered: Among multiple forms of cell death, which one hold the predominate responsibility for the development of different bone diseases? How to effectively block the pro-osteoclastogenic effects of osteocyte death and how to translate into clinical application? It has been revealed that osteocyte death exerts regulatory effects on osteoclast formation, but some mechanisms remain unclear and require more studies to explore. Meanwhile, some novel forms of cell death are recently revealed, such as cuproptosis, and might participate in bone remodeling under specific conditions (165). In addition, various forms of cell death do not act in an isolate way, but interact with each other. Thus, a novel concept of integrated cell death emerges and are named as PANoptosis, which refers to the cell death modality involving the interactions among pyroptosis, apoptosis, and necroptosis (166). Besides, studies also revealed the interaction between ferroptosis and other cell deaths, such as apoptosis and pyroptosis (149). Together, there might be an intracellular network composed of various death signaling pathways which is activated under pathological conditions. A comprehensive understanding of cell death in osteocytes and their role in osteoclast regulation is needed and requires more studies to reveal.

## References

[1] Florencio-Silva R Sasso GR Sasso-Cerri E Simões MJ Cerri PS. Biology of bone tissue: structure, function, and factors that influence bone cells. BioMed Res Int. (2015) 2015:421746. doi: 10.1155/2015/421746 26247020 PMC4515490

[2] Durdan MM Azaria RD Weivoda MM. Novel insights into the coupling of osteoclasts and resorption to bone formation. Semin Cell Dev Biol. (2022) 123:4–13. doi: 10.1016/j.semcdb.2021.10.008 34756783 PMC8840962

[3] Gao L Liu G Wu X Liu C Wang Y Ma M. Osteocytes autophagy mediated by mTORC2 activation controls osteoblasts differentiation and osteoclasts activities under mechanical loading. Arch Biochem Biophys. (2023) 742:109634. doi: 10.1016/j.abb.2023.109634 37164247

[4] Chen H Senda T Kubo KY. The osteocyte plays multiple roles in bone remodeling and mineral homeostasis. Med Mol Morphol. (2015) 48:61–8. doi: 10.1007/s00795-015-0099-y 25791218

[5] Tresguerres FGF Torres J López-Quiles J Hernández G Vega JA Tresguerres IF. The osteocyte: A multifunctional cell within the bone. Ann Anat. (2020) 227:151422. doi: 10.1016/j.aanat.2019.151422 31563568

[6] Boyle WJ Simonet WS Lacey DL. Osteoclast differentiation and activation. Nature. (2003) 423:337–42. doi: 10.1038/nature01658 12748652

[7] Geissmann F Manz MG Jung S Sieweke MH Merad M Ley K. Development of monocytes, macrophages, and dendritic cells. Science. (2010) 327:656–61. doi: 10.1126/science.1178331 PMC2887389 20133564

[8] Tsukasaki M Takayanagi H. Osteoimmunology: evolving concepts in bone-immune interactions in health and disease. Nat Rev Immunol. (2019) 19:626–42. doi: 10.1038/s41577-019-0178-8 31186549

[9] Boyce BF Xing L. Functions of RANKL/RANK/OPG in bone modeling and remodeling. Arch Biochem Biophys. (2008) 473:139–46. doi: 10.1016/j.abb.2008.03.018 PMC2413418 18395508

[10] Xiong J Onal M Jilka RL Weinstein RS Manolagas SC O’Brien CA. Matrix-embedded cells control osteoclast formation. Nat Med. (2011) 17:1235–41. doi: 10.1038/nm.2448 PMC3192296 21909103

[11] Nakashima T Hayashi M Fukunaga T Kurata K Oh-Hora M Feng JQ. Evidence for osteocyte regulation of bone homeostasis through RANKL expression. Nat Med. (2011) 17:1231–4. doi: 10.1038/nm.2452 21909105

[12] Gu G Mulari M Peng Z Hentunen TA Vaananen HK. Death of osteocytes turns off the inhibition of osteoclasts and triggers local bone resorption. Biochem Biophys Res Commun. (2005) 335:1095–101. doi: 10.1016/j.bbrc.2005.06.211 16111656

[13] Zhou Z Han JY Xi CX Xie JX Feng X Wang CY. HMGB1 regulates RANKL-induced osteoclastogenesis in a manner dependent on RAGE. J Bone Miner Res. (2008) 23:1084–96. doi: 10.1359/jbmr.080234 PMC2679382 18302500

[14] Kogianni G Mann V Noble BS. Apoptotic bodies convey activity capable of initiating osteoclastogenesis and localized bone destruction. J Bone Miner Res. (2008) 23:915–27. doi: 10.1359/jbmr.080207 18435576

[15] Cui H Li J Li X Su T Wen P Wang C. TNF-α promotes osteocyte necroptosis by upregulating TLR4 in postmenopausal osteoporosis. Bone. (2024) 182:117050. doi: 10.1016/j.bone.2024.117050 38367924

[16] Jiang Z Qi G He X Yu Y Cao Y Zhang C. Ferroptosis in osteocytes as a target for protection against postmenopausal osteoporosis. Adv Sci (Weinh). (2024) 11:e2307388. doi: 10.1002/advs.202307388 38233202 PMC10966575

[17] Speziani C Rivollier A Gallois A Coury F Mazzorana M Azocar O. Murine dendritic cell transdifferentiation into osteoclasts is differentially regulated by innate and adaptive cytokines. Eur J Immunol. (2007) 37:747–57. doi: 10.1002/eji.200636534 17304626

[18] Yao Z Getting SJ Locke IC. Regulation of TNF-induced osteoclast differentiation. Cells. (2021) 11:132. doi: 10.3390/cells11010132 35011694 PMC8750957

[19] Stenbeck G. Formation and function of the ruffled border in osteoclasts. Semin Cell Dev Biol. (2002) 13:285–92. doi: 10.1016/S1084952102000587 12243728

[20] Gong T Liu L Jiang W Zhou R. DAMP-sensing receptors in sterile inflammation and inflammatory diseases. Nat Rev Immunol. (2019) 20:95–112. doi: 10.1038/s41577-019-0215-7 31558839

[21] Wyczanska M Lange-Sperandio B. DAMPs in unilateral ureteral obstruction. Front Immunol. (2020) 11:581300. doi: 10.3389/fimmu.2020.581300 33117389 PMC7575708

[22] Lin S-Y Hsieh S-Y Fan Y-T Wei W-C Hsiao P-W Tsai D-H. Necroptosis promotes autophagy-dependent upregulation of DAMP and results in immunosurveillance. Autophagy. (2017) 14:778–95. doi: 10.1080/15548627.2017.1386359 PMC6070008 29171784

[23] Amarante-Mendes GP Adjemian S Branco LM Zanetti LC Weinlich R Bortoluci KR. Pattern recognition receptors and the host cell death molecular machinery. Front Immunol. (2018) 9:2379. doi: 10.3389/fimmu.2018.02379 30459758 PMC6232773

[24] Kong Y-Y Feige U Sarosi I Bolon B Tafuri A Morony S. Activated T cells regulate bone loss and joint destruction in adjuvant arthritis through osteoprotegerin ligand. Nature. (1999) 402:304–9. doi: 10.1038/46303 10580503

[25] Belibasakis GN Bostanci N Hashim A Johansson A Aduse-Opoku J Curtis MA. Regulation of RANKL and OPG gene expression in human gingival fibroblasts and periodontal ligament cells by Porphyromonas gingivalis: A putative role of the Arg-gingipains. Microb Pathog. (2007) 43:46–53. doi: 10.1016/j.micpath.2007.03.001 17448630

[26] Kanematsu M Sato T Takai H Watanabe K Ikeda K Yamada Y. Prostaglandin E2 induces expression of receptor activator of nuclear factor-kappa B ligand/osteoprotegrin ligand on pre-B cells: implications for accelerated osteoclastogenesis in estrogen deficiency. J Bone Miner Res. (2000) 15:1321–9. doi: 10.1359/jbmr.2000.15.7.1321 10893680

[27] Yamashita T Yao Z Li F Zhang Q Badell IR Schwarz EM. NF-κB p50 and p52 regulate receptor activator of NF-κB ligand (RANKL) and tumor necrosis factor-induced osteoclast precursor differentiation by activating c-Fos and NFATc1. J Biol Chem. (2007) 282:18245–53. doi: 10.1074/jbc.M610701200 17485464

[28] Takayanagi H Kim S Koga T Nishina H Isshiki M Yoshida H. Induction and activation of the transcription factor NFATc1 (NFAT2) integrate RANKL signaling in terminal differentiation of osteoclasts. Dev Cell. (2002) 3:889–901. doi: 10.1016/S1534-5807(02)00369-6 12479813

[29] Takayanagi H. The role of NFAT in osteoclast formation. Ann N Y Acad Sci. (2007) 1116:227–37. doi: 10.1196/annals.1402.071 18083930

[30] Manabe N Kawaguchi H Chikuda H Miyaura C Inada M Nagai R. Connection between B lymphocyte and osteoclast differentiation pathways. J Immunol. (2001) 167:2625–31. doi: 10.4049/jimmunol.167.5.2625 11509604

[31] Pugliese LS Gonçalves TO Popi AF Mariano M Pesquero JB Lopes JD. B-1 lymphocytes differentiate into functional osteoclast-like cells. Immunobiology. (2012) 217:336–44. doi: 10.1016/j.imbio.2011.07.014 21855167

[32] Sato T Shibata T Ikeda K Watanabe K. Generation of bone-resorbing osteoclasts from B220+ Cells: its role in accelerated osteoclastogenesis due to estrogen deficiency. J Bone Miner Res. (2001) 16:2215–21. doi: 10.1359/jbmr.2001.16.12.2215 11760834

[33] Boyce BF Xing L. Biology of RANK, RANKL, and osteoprotegerin. Arthritis Res Ther. (2007) 9:S1. doi: 10.1186/ar2165 17634140 PMC1924516

[34] Bucay N Sarosi I Dunstan CR Morony S Tarpley J Capparelli C. Osteoprotegerin-deficient mice develop early onset osteoporosis and arterial calcification. Genes Dev. (1998) 12:1260–8. doi: 10.1101/gad.12.9.1260 PMC316769 9573043

[35] Simonet WS Lacey DL Dunstan CR Kelley M Chang MS Lüthy R. Osteoprotegerin: A novel secreted protein involved in the regulation of bone density. Cell. (1997) 89:309–19. doi: 10.1016/S0092-8674(00)80209-3 9108485

[36] Whyte MP Obrecht SE Finnegan PM Jones JL Podgornik MN McAlister WH. Osteoprotegerin deficiency and juvenile Paget’s disease. N Engl J Med. (2002) 347:175–84. doi: 10.1056/NEJMoa013096 12124406

[37] Dostert C Grusdat M Letellier E Brenner D. The TNF family of ligands and receptors: communication modules in the immune system and beyond. Physiol Rev. (2019) 99:115–60. doi: 10.1152/physrev.00045.2017 30354964

[38] Zha L He L Liang Y Qin H Yu B Chang L. TNF-α contributes to postmenopausal osteoporosis by synergistically promoting RANKL-induced osteoclast formation. BioMed Pharmacother. (2018) 102:369–74. doi: 10.1016/j.biopha.2018.03.080 29571022

[39] Marahleh A Kitaura H Ohori F Kishikawa A Ogawa S Shen WR. TNF-α Directly enhances osteocyte RANKL expression and promotes osteoclast formation. Front Immunol. (2019) 10:2925. doi: 10.3389/fimmu.2019.02925 31921183 PMC6923682

[40] Ohori F Kitaura H Marahleh A Kishikawa A Ogawa S Qi J. Effect of TNF-α-induced sclerostin on osteocytes during orthodontic tooth movement. J Immunol Res. (2019) 2019:9716758. doi: 10.1155/2019/9716758 31341915 PMC6612957

[41] Zhao Z Hou X Yin X Li Y Duan R Boyce BF. TNF induction of NF-κB RelB enhances RANKL-induced osteoclastogenesis by promoting inflammatory macrophage differentiation but also limits it through suppression of NFATc1 expression. PloS One. (2015) 10:e0135728. doi: 10.1371/journal.pone.0135728 26287732 PMC4545392

[42] Li J Sarosi I Yan XQ Morony S Capparelli C Tan HL. RANK is the intrinsic hematopoietic cell surface receptor that controls osteoclastogenesis and regulation of bone mass and calcium metabolism. Proc Natl Acad Sci U.S.A. (2000) 97:1566–71. doi: 10.1073/pnas.97.4.1566 PMC26475 10677500

[43] Zhao B Grimes SN Li S Hu X Ivashkiv LB. TNF-induced osteoclastogenesis and inflammatory bone resorption are inhibited by transcription factor RBP-J. J Exp Med. (2012) 209:319–34. doi: 10.1084/jem.20111566 PMC3280875 22249448

[44] O’Brien W Fissel BM Maeda Y Yan J Ge X Gravallese EM. RANK-independent osteoclast formation and bone erosion in inflammatory arthritis. Arthritis Rheumatol. (2016) 68:2889–900. doi: 10.1002/art.39837 PMC5125876 27563728

[45] Claude-Taupin A Bissa B Jia J Gu Y Deretic V. Role of autophagy in IL-1β export and release from cells. Semin Cell Dev Biol. (2018) 83:36–41. doi: 10.1016/j.semcdb.2018.03.012 29580970 PMC6173661

[46] Almehmadi AH Alghamdi F. Biomarkers of alveolar bone resorption in gingival crevicular fluid: A systematic review. Arch Oral Biol. (2018) 93:12–21. doi: 10.1016/j.archoralbio.2018.05.004 29800801

[47] McLean RR. Proinflammatory cytokines and osteoporosis. Curr Osteoporos Rep. (2009) 7:134–9. doi: 10.1007/s11914-009-0023-2 19968917

[48] Dinarello CA. The IL-1 family of cytokines and receptors in rheumatic diseases. Nat Rev Rheumatol. (2019) 15:612–32. doi: 10.1038/s41584-019-0277-8 31515542

[49] Kulkarni RN Bakker AD Everts V Klein-Nulend J. Mechanical loading prevents the stimulating effect of IL-1β on osteocyte-modulated osteoclastogenesis. Biochem Biophys Res Commun. (2012) 420:11–6. doi: 10.1016/j.bbrc.2012.02.099 22390927

[50] Pathak JL Bakker AD Luyten FP Verschueren P Lems WF Klein-Nulend J. Systemic inflammation affects human osteocyte-specific protein and cytokine expression. Calcif Tissue Int. (2016) 98:596–608. doi: 10.1007/s00223-016-0116-8 26887974

[51] Robaszkiewicz A Qu C Wisnik E Ploszaj T Mirsaidi A Kunze FA. ARTD1 regulates osteoclastogenesis and bone homeostasis by dampening NF-κB-dependent transcription of IL-1β. Sci Rep. (2016) 6:21131. doi: 10.1038/srep21131 26883084 PMC4756713

[52] Lee J Park C Kim HJ Lee YD Lee ZH Song YW. Stimulation of osteoclast migration and bone resorption by c–c chemokine ligands 19 and 21. Exp Mol Med. (2017) 49:e358. doi: 10.1038/emm.2017.100 28729639 PMC5565950

[53] Panagakos FS Jandinski JJ Feder L Kumar S. Effects of plasminogen and interleukin-1β on bone resorption *in vitro* . Biochimie. (1994) 76:394–7. doi: 10.1016/0300-9084(94)90114-7 7849104

[54] Uciechowski P Dempke WCM. Interleukin-6: A masterplayer in the cytokine network. Oncology. (2020) 98:131–7. doi: 10.1159/000505099 31958792

[55] Cheung WY Simmons CA You L. Osteocyte apoptosis regulates osteoclast precursor adhesion via osteocytic IL-6 secretion and endothelial ICAM-1 expression. Bone. (2012) 50:104–10. doi: 10.1016/j.bone.2011.09.052 21986000

[56] Yao X Huang J Zhong H Shen N Faggioni R Fung M. Targeting interleukin-6 in inflammatory autoimmune diseases and cancers. Pharmacol Ther. (2014) 141:125–39. doi: 10.1016/j.pharmthera.2013.09.004 24076269

[57] Choy EH De Benedetti F Takeuchi T Hashizume M John MR Kishimoto T. Translating IL-6 biology into effective treatments. Nat Rev Rheumatol. (2020) 16:335–45. doi: 10.1038/s41584-020-0419-z PMC7178926 32327746

[58] Tanaka K Hashizume M Mihara M Yoshida H Suzuki M Matsumoto Y. Anti-interleukin-6 receptor antibody prevents systemic bone mass loss via reducing the number of osteoclast precursors in bone marrow in a collagen-induced arthritis model. Clin Exp Immunol. (2014) 175:172–80. doi: 10.1111/cei.12201 PMC3892408 24028747

[59] Udagawa N Takahashi N Katagiri T Tamura T Wada S Findlay DM. Interleukin (IL)-6 induction of osteoclast differentiation depends on IL-6 receptors expressed on osteoblastic cells but not on osteoclast progenitors. J Exp Med. (1995) 182:1461–8. doi: 10.1084/jem.182.5.1461 PMC2192181 7595216

[60] Wang T He C. TNF-α and IL-6: The link between immune and bone system. Curr Drug Targets. (2020) 21:213–27. doi: 10.2174/1389450120666190821161259 31433756

[61] O’Brien CA Gubrij I Lin SC Saylors RL Manolagas SC. STAT3 activation in stromal/osteoblastic cells is required for induction of the receptor activator of NF-kappaB ligand and stimulation of osteoclastogenesis by gp130-utilizing cytokines or interleukin-1 but not 1,25-dihydroxyvitamin D3 or parathyroid hormone. J Biol Chem. (1999) 274:19301–8. doi: 10.1074/jbc.274.27.19301 10383440

[62] Wang JS Mazur CM Wein MN. Sclerostin and osteocalcin: candidate bone-produced hormones. Front Endocrinol (Lausanne). (2021) 12:584147. doi: 10.3389/fendo.2021.584147 33776907 PMC7988212

[63] Wijenayaka AR Kogawa M Lim HP Bonewald LF Findlay DM Atkins GJ. Sclerostin stimulates osteocyte support of osteoclast activity by a RANKL-dependent pathway. PloS One. (2011) 6:e25900. doi: 10.1371/journal.pone.0025900 21991382 PMC3186800

[64] Shaw P Dwivedi SKD Bhattacharya R Mukherjee P Rao G. VEGF signaling: Role in angiogenesis and beyond. Biochim Biophys Acta Rev Cancer. (2024) 1879:189079. doi: 10.1016/j.bbcan.2024.189079 38280470 PMC12927493

[65] Jilka RL Noble B Weinstein RS. Osteocyte apoptosis. Bone. (2013) 54:264–71. doi: 10.1016/j.bone.2012.11.038 PMC3624050 23238124

[66] Niida S Kaku M Amano H Yoshida H Kataoka H Nishikawa S. Vascular endothelial growth factor can substitute for macrophage colony-stimulating factor in the support of osteoclastic bone resorption. J Exp Med. (1999) 190:293–8. doi: 10.1084/jem.190.2.293 PMC2195572 10432291

[67] Yang Q McHugh KP Patntirapong S Gu X Wunderlich L Hauschka PV. VEGF enhancement of osteoclast survival and bone resorption involves VEGF receptor-2 signaling and beta3-integrin. Matrix Biol. (2008) 27:589–99. doi: 10.1016/j.matbio.2008.06.005 18640270

[68] Charoonpatrapong K Shah R Robling AG Alvarez M Clapp DW Chen S. HMGB1 expression and release by bone cells. J Cell Physiol. (2006) 207:480–90. doi: 10.1002/jcp.20577 16419037

[69] Yang J Shah R Robling AG Templeton E Yang H Tracey KJ. HMGB1 is a bone-active cytokine. J Cell Physiol. (2008) 214:730–9. doi: 10.1002/jcp.21268 17786958

[70] Scaffidi P Misteli T Bianchi ME. Release of chromatin protein HMGB1 by necrotic cells triggers inflammation. Nature. (2002) 418:191–5. doi: 10.1038/nature00858 12110890

[71] Bidwell JP Yang J Robling AG. Is HMGB1 an osteocyte alarmin? J Cell Biochem. (2008) 103:1671–80. doi: 10.1002/jcb.21572 17948903

[72] Davis HM Valdez S Gomez L Malicky P White FA Subler MA. High mobility group box 1 protein regulates osteoclastogenesis through direct actions on osteocytes and osteoclasts *in vitro* . J Cell Biochem. (2019) 120:16741–9. doi: 10.1002/jcb.28932 PMC6713577 31106449

[73] Wang W Zhang H Sandai D Zhao R Bai J Wang Y. ATP-induced cell death: a novel hypothesis for osteoporosis. Front Cell Dev Biol. (2023) 11:1324213. doi: 10.3389/fcell.2023.1324213 38161333 PMC10755924

[74] Kringelbach TM Aslan D Novak I Schwarz P Jørgensen NR. UTP-induced ATP release is a fine-tuned signalling pathway in osteocytes. Purinergic Signal. (2014) 10:337–47. doi: 10.1007/s11302-013-9404-1 PMC4040174 24374572

[75] Lu J Shi X Fu Q Han Y Zhu L Zhou Z. New mechanistic understanding of osteoclast differentiation and bone resorption mediated by P2X7 receptors and PI3K-Akt-GSK3β signaling. Cell Mol Biol Lett. (2024) 29:100. doi: 10.1186/s11658-024-00614-5 38977961 PMC11232284

[76] Gartland A Buckley KA Bowler WB Gallagher JA. Blockade of the pore-forming P2X7 receptor inhibits formation of multinucleated human osteoclasts *in vitro* . Calcif Tissue Int. (2003) 73:361–9. doi: 10.1007/s00223-002-2098-y 12874700

[77] Hazama R Qu X Yokoyama K Tanaka C Kinoshita E He J. ATP-induced osteoclast function: The formation of sealing-zone like structure and the secretion of lytic granules via microtubule-deacetylation under the control of Syk. Genes Cells. (2009) 14:871–84. doi: 10.1111/j.1365-2443.2009.01317.x 19549171

[78] Miyazaki T Iwasawa M Nakashima T Mori S Shigemoto K Nakamura H. Intracellular and extracellular ATP coordinately regulate the inverse correlation between osteoclast survival and bone resorption. J Biol Chem. (2012) 287:37808–23. doi: 10.1074/jbc.M112.385369 PMC3488055 22988253

[79] Morrison MS Turin L King BF Burnstock G Arnett TR. ATP is a potent stimulator of the activation and formation of rodent osteoclasts. J Physiol. (1998) 511:495–500. doi: 10.1111/j.1469-7793.1998.495bh.x 9706025 PMC2231120

[80] Kroemer G El-Deiry WS Golstein P Peter ME Vaux D Vandenabeele P. Classification of cell death: recommendations of the Nomenclature Committee on Cell Death. Cell Death Differ. (2005) 12 Suppl 2:1463–7. doi: 10.1038/sj.cdd.4401724 16247491

[81] Galluzzi L Maiuri MC Vitale I Zischka H Castedo M Zitvogel L. Cell death modalities: classification and pathophysiological implications. Cell Death Differ. (2007) 14:1237–43. doi: 10.1038/sj.cdd.4402148 17431418

[82] Newton K Strasser A Kayagaki N Dixit VM. Cell death. Cell. (2024) 187:235–56. doi: 10.1016/j.cell.2023.11.044 38242081

[83] Leist M Jäättelä M. Four deaths and a funeral: from caspases to alternative mechanisms. Nat Rev Mol Cell Biol. (2001) 2:589–98. doi: 10.1038/35085008 11483992

[84] Tower J. Programmed cell death in aging. Ageing Res Rev. (2015) 23:90–100. doi: 10.1016/j.arr.2015.04.002 25862945 PMC4480161

[85] Tong X Tang R Xiao M Xu J Wang W Zhang B. Targeting cell death pathways for cancer therapy: recent developments in necroptosis, pyroptosis, ferroptosis, and cuproptosis research. J Hematol Oncol. (2022) 15:174. doi: 10.1186/s13045-022-01392-3 36482419 PMC9733270

[86] Liu S Pan Y Li T Zou M Liu W Li Q. The role of regulated programmed cell death in osteoarthritis: from pathogenesis to therapy. Int J Mol Sci. (2023) 24:5364. doi: 10.3390/ijms24065364 36982438 PMC10049357

[87] Al-Dujaili SA Lau E Al-Dujaili H Tsang K Guenther A You L. Apoptotic osteocytes regulate osteoclast precursor recruitment and differentiation *in vitro* . J Cell Biochem. (2011) 112:2412–23. doi: 10.1002/jcb.23164 21538477

[88] Zong WX Thompson CB. Necrotic death as a cell fate. Genes Dev. (2006) 20:1–15. doi: 10.1101/gad.1376506 16391229

[89] Andreev D Liu M Weidner D Kachler K Faas M Grüneboom A. Osteocyte necrosis triggers osteoclast-mediated bone loss through macrophage-inducible C-type lectin. J Clin Invest. (2020) 130:4811–30. doi: 10.1172/jci134214 PMC7456234 32773408

[90] Gkouveris I Hadaya D Elzakra N Soundia A Bezouglaia O Dry SM. Inhibition of HMGB1/RAGE signaling reduces the incidence of medication-related osteonecrosis of the jaw (MRONJ) in mice. J Bone Miner Res. (2022) 37:1775–86. doi: 10.1002/jbmr.4637 PMC9474692 35711109

[91] Komori T. Cell death in chondrocytes, osteoblasts, and osteocytes. Int J Mol Sci. (2016) 17:2045. doi: 10.3390/ijms17122045 27929439 PMC5187845

[92] Quan H Ren C He Y Wang F Dong S Jiang H. Application of biomaterials in treating early osteonecrosis of the femoral head: Research progress and future perspectives. Acta Biomater. (2023) 164:15–73. doi: 10.1016/j.actbio.2023.04.005 37080444

[93] Chen K Liu Y He J Pavlos N Wang C Kenny J. Steroid-induced osteonecrosis of the femoral head reveals enhanced reactive oxygen species and hyperactive osteoclasts. Int J Biol Sci. (2020) 16:1888–900. doi: 10.7150/ijbs.40917 PMC7211180 32398957

[94] Varoga D Drescher W Pufe M Groth G Pufe T. Differential expression of vascular endothelial growth factor in glucocorticoid-related osteonecrosis of the femoral head. Clin Orthop Relat Res. (2009) 467:3273–82. doi: 10.1007/s11999-009-1076-3 PMC2772917 19763724

[95] Huang J Ma T Wang C Wang Z Wang X Hua B. SOST/Sclerostin impairs the osteogenesis and angiogesis in glucocorticoid-associated osteonecrosis of femoral head. Mol Med. (2024) 30:167. doi: 10.1186/s10020-024-00933-5 39342093 PMC11439244

[96] Kroemer G Galluzzi L Brenner C. Mitochondrial membrane permeabilization in cell death. Physiol Rev. (2007) 87:99–163. doi: 10.1152/physrev.00013.2006 17237344

[97] Dewson G Ma S Frederick P Hockings C Tan I Kratina T. Bax dimerizes via a symmetric BH3:groove interface during apoptosis. Cell Death Differ. (2012) 19:661–70. doi: 10.1038/cdd.2011.138 PMC3307980 22015607

[98] Kumar S. Caspase function in programmed cell death. Cell Death Differ. (2007) 14:32–43. doi: 10.1038/sj.cdd.4402060 17082813

[99] Fischer U Jänicke RU Schulze-Osthoff K. Many cuts to ruin: a comprehensive update of caspase substrates. Cell Death Differ. (2003) 10:76–100. doi: 10.1038/sj.cdd.4401160 12655297 PMC7091709

[100] Tian X Srinivasan PR Tajiknia V Sanchez Sevilla Uruchurtu AF Seyhan AA Carneiro BA. Targeting apoptotic pathways for cancer therapy. J Clin Invest. (2024) 134:e179570. doi: 10.1172/jci179570 39007268 PMC11245162

[101] Xiao L Zhang L Guo C Xin Q Gu X Jiang C. Find Me” and “Eat Me” signals: tools to drive phagocytic processes for modulating antitumor immunity. Cancer Commun (Lond). (2024) 44:791–832. doi: 10.1002/cac2.12579 38923737 PMC11260773

[102] Figueiredo PA Powers SK Ferreira RM Appell HJ Duarte JA. Aging impairs skeletal muscle mitochondrial bioenergetic function. J Gerontol A Biol Sci Med Sci. (2009) 64:21–33. doi: 10.1093/gerona/gln048 19196905 PMC2691197

[103] Kogianni G Mann V Ebetino F Nuttall M Nijweide P Simpson H. Fas/CD95 is associated with glucocorticoid-induced osteocyte apoptosis. Life Sci. (2004) 75:2879–95. doi: 10.1016/j.lfs.2004.04.048 15454340

[104] Almeida M Laurent MR Dubois V Claessens F O’Brien CA Bouillon R. Estrogens and androgens in skeletal physiology and pathophysiology. Physiol Rev. (2017) 97:135–87. doi: 10.1152/physrev.00033.2015 PMC5539371 27807202

[105] Wu X Feng X He Y Gao Y Yang S Shao Z. IL-4 administration exerts preventive effects via suppression of underlying inflammation and TNF-α-induced apoptosis in steroid-induced osteonecrosis. Osteoporos Int. (2016) 27:1827–37. doi: 10.1007/s00198-015-3474-6 26753542

[106] Mann V Huber C Kogianni G Jones D Noble B. The influence of mechanical stimulation on osteocyte apoptosis and bone viability in human trabecular bone. J Musculoskelet Neuronal Interact. (2006) 6:408–17. PMC1847464 17185839

[107] You LD Weinbaum S Cowin SC Schaffler MB. Ultrastructure of the osteocyte process and its pericellular matrix. Anat Rec A Discovery Mol Cell Evol Biol. (2004) 278:505–13. doi: 10.1002/ar.a.20050 15164337

[108] Aguirre JI Plotkin LI Stewart SA Weinstein RS Parfitt AM Manolagas SC. Osteocyte apoptosis is induced by weightlessness in mice and precedes osteoclast recruitment and bone loss. J Bone Miner Res. (2006) 21:605–15. doi: 10.1359/jbmr.060107 16598381

[109] Chen H Liu W Wu X Gou M Shen J Wang H. Advanced glycation end products induced IL-6 and VEGF-A production and apoptosis in osteocyte-like MLO-Y4 cells by activating RAGE and ERK1/2, P38 and STAT3 signalling pathways. Int Immunopharmacol. (2017) 52:143–9. doi: 10.1016/j.intimp.2017.09.004 28910744

[110] Cabahug-Zuckerman P Frikha-Benayed D Majeska RJ Tuthill A Yakar S Judex S. Osteocyte apoptosis caused by hindlimb unloading is required to trigger osteocyte RANKL production and subsequent resorption of cortical and trabecular bone in mice femurs. J Bone Miner Res. (2016) 31:1356–65. doi: 10.1002/jbmr.2807 PMC5488280 26852281

[111] Kitaura H Marahleh A Ohori F Noguchi T Shen WR Qi J. Osteocyte-related cytokines regulate osteoclast formation and bone resorption. Int J Mol Sci. (2020) 21:5169. doi: 10.3390/ijms21145169 32708317 PMC7404053

[112] Emerton KB Hu B Woo AA Sinofsky A Hernandez C Majeska RJ. Osteocyte apoptosis and control of bone resorption following ovariectomy in mice. Bone. (2010) 46:577–83. doi: 10.1016/j.bone.2009.11.006 PMC2824001 19925896

[113] Verborgt O Tatton NA Majeska RJ Schaffler MB. Spatial distribution of Bax and Bcl-2 in osteocytes after bone fatigue: complementary roles in bone remodeling regulation? J Bone Miner Res. (2002) 17:907–14. doi: 10.1359/jbmr.2002.17.5.907 12009022

[114] Cheung WY Fritton JC Morgan SA Seref-Ferlengez Z Basta-Pljakic J Thi MM. Pannexin-1 and P2X7-receptor are required for apoptotic osteocytes in fatigued bone to trigger RANKL production in neighboring bystander osteocytes. J Bone Miner Res. (2016) 31:890–9. doi: 10.1002/jbmr.2740 PMC4915221 26553756

[115] Takemura Y Moriyama Y Ayukawa Y Kurata K Rakhmatia YD Koyano K. Mechanical loading induced osteocyte apoptosis and connexin 43 expression in three-dimensional cell culture and dental implant model. J BioMed Mater Res A. (2019) 107:815–27. doi: 10.1002/jbm.a.36597 30578719

[116] Messmer D Yang H Telusma G Knoll F Li J Messmer B. High mobility group box protein 1: an endogenous signal for dendritic cell maturation and Th1 polarization. J Immunol. (2004) 173:307–13. doi: 10.4049/jimmunol.173.1.307 15210788

[117] Plotkin LI Gortazar AR Davis HM Condon KW Gabilondo H Maycas M. Inhibition of osteocyte apoptosis prevents the increase in osteocytic receptor activator of nuclear factor κB ligand (RANKL) but does not stop bone resorption or the loss of bone induced by unloading. J Biol Chem. (2015) 290:18934–42. doi: 10.1074/jbc.M115.642090 PMC4521013 26085098

[118] He B Zhu Y Cui H Sun B Su T Wen P. Comparison of necroptosis with apoptosis for OVX-induced osteoporosis. Front Mol Biosci. (2021) 8:790613. doi: 10.3389/fmolb.2021.790613 35004853 PMC8740137

[119] Degterev A Huang Z Boyce M Li Y Jagtap P Mizushima N. Chemical inhibitor of nonapoptotic cell death with therapeutic potential for ischemic brain injury. Nat Chem Biol. (2005) 1:112–9. doi: 10.1038/nchembio711 16408008

[120] Seo J Nam YW Kim S Oh DB Song J. Necroptosis molecular mechanisms: Recent findings regarding novel necroptosis regulators. Exp Mol Med. (2021) 53:1007–17. doi: 10.1038/s12276-021-00634-7 PMC8166896 34075202

[121] Lork M Verhelst K Beyaert R. CYLD. A20 and OTULIN deubiquitinases in NF-κB signaling and cell death: so similar, yet so different. Cell Death Differ. (2017) 24:1172–83. doi: 10.1038/cdd.2017.46 PMC5520167 28362430

[122] Someda M Kuroki S Miyachi H Tachibana M Yonehara S. Caspase-8, receptor-interacting protein kinase 1 (RIPK1), and RIPK3 regulate retinoic acid-induced cell differentiation and necroptosis. Cell Death Differ. (2020) 27:1539–53. doi: 10.1038/s41418-019-0434-2 PMC7206185 31659279

[123] Zhang Y Liu J Yu D Zhu X Liu X Liao J. The MLKL kinase-like domain dimerization is an indispensable step of mammalian MLKL activation in necroptosis signaling. Cell Death Dis. (2021) 12:638. doi: 10.1038/s41419-021-03859-6 34158471 PMC8219780

[124] Hu X Wang Z Kong C Wang Y Zhu W Wang W. Necroptosis: A new target for prevention of osteoporosis. Front Endocrinol (Lausanne). (2022) 13:1032614. doi: 10.3389/fendo.2022.1032614 36339402 PMC9627214

[125] Xue F Zhao Z Gu Y Han J Ye K Zhang Y. 7,8-Dihydroxyflavone modulates bone formation and resorption and ameliorates ovariectomy-induced osteoporosis. Elife. (2021) 10:e64872. doi: 10.7554/eLife.64872 34227467 PMC8285109

[126] Murao A Aziz M Wang H Brenner M Wang P. Release mechanisms of major DAMPs. Apoptosis. (2021) 26:152–62. doi: 10.1007/s10495-021-01663-3 PMC8016797 33713214

[127] Zhu YM Lin L Wei C Guo Y Qin Y Li ZS. The key regulator of necroptosis, RIP1 kinase, contributes to the formation of astrogliosis and glial scar in ischemic stroke. Transl Stroke Res. (2021) 12:991–1017. doi: 10.1007/s12975-021-00888-3 33629276 PMC8557200

[128] Cui H Zhu Y Yang Q Zhao W Zhang S Zhou A. Necrostatin-1 treatment inhibits osteocyte necroptosis and trabecular deterioration in ovariectomized rats. Sci Rep. (2016) 6:33803. doi: 10.1038/srep33803 27703177 PMC5050438

[129] Feng M Zhang R Gong F Yang P Fan L Ni J. Protective effects of necrostatin-1 on glucocorticoid-induced osteoporosis in rats. J Steroid Biochem Mol Biol. (2014) 144 Pt B:455–62. doi: 10.1016/j.jsbmb.2014.09.005 25220755

[130] Liu P Wang W Li Z Li Y Yu X Tu J. Ferroptosis: A new regulatory mechanism in osteoporosis. Oxid Med Cell Longev. (2022) 2022:2634431. doi: 10.1155/2022/2634431 35082963 PMC8786466

[131] Stockwell BR. Ferroptosis turns 10: Emerging mechanisms, physiological functions, and therapeutic applications. Cell. (2022) 185:2401–21. doi: 10.1016/j.cell.2022.06.003 PMC9273022 35803244

[132] Yan C Zhang J An F Wang J Shi Y Yuan L. Research progress of ferroptosis regulatory network and bone remodeling in osteoporosis. Front Public Health. (2022) 10:910675. doi: 10.3389/fpubh.2022.910675 35844870 PMC9280046

[133] Sun S Shen J Jiang J Wang F Min J. Targeting ferroptosis opens new avenues for the development of novel therapeutics. Signal Transduct Target Ther. (2023) 8:372. doi: 10.1038/s41392-023-01606-1 37735472 PMC10514338

[134] Henning Y Blind US Larafa S Matschke J Fandrey J. Hypoxia aggravates ferroptosis in RPE cells by promoting the Fenton reaction. Cell Death Dis. (2022) 13:662. doi: 10.1038/s41419-022-05121-z 35906211 PMC9338085

[135] He YJ Liu XY Xing L Wan X Chang X Jiang HL. Fenton reaction-independent ferroptosis therapy via glutathione and iron redox couple sequentially triggered lipid peroxide generator. Biomaterials. (2020) 241:119911. doi: 10.1016/j.biomaterials.2020.119911 32143060

[136] Ru Q Li Y Chen L Wu Y Min J Wang F. Iron homeostasis and ferroptosis in human diseases: mechanisms and therapeutic prospects. Signal Transduct Target Ther. (2024) 9:271. doi: 10.1038/s41392-024-01969-z 39396974 PMC11486532

[137] Wu X Fang X Lu F Chen Q Liu J Zheng L. An update on the role of ferroptosis in the pathogenesis of osteoporosis. EFORT Open Rev. (2024) 9:712–22. doi: 10.1530/eor-23-0148 PMC11370720 39087516

[138] Parker JL Deme JC Kolokouris D Kuteyi G Biggin PC Lea SM. Molecular basis for redox control by the human cystine/glutamate antiporter system xc(). Nat Commun. (2021) 12:7147. doi: 10.1038/s41467-021-27414-1 34880232 PMC8654953

[139] Gan B. How erastin assassinates cells by ferroptosis revealed. Protein Cell. (2023) 14:84–6. doi: 10.1093/procel/pwac007 PMC10019563 36929006

[140] Yang J Dong D Luo X Zhou J Shang P Zhang H. Iron overload-induced osteocyte apoptosis stimulates osteoclast differentiation through increasing osteocytic RANKL production *in vitro* . Calcif Tissue Int. (2020) 107:499–509. doi: 10.1007/s00223-020-00735-x 32995951

[141] Ma J Wang A Zhang H Liu B Geng Y Xu Y. Iron overload induced osteocytes apoptosis and led to bone loss in Hepcidin(-/-) mice through increasing sclerostin and RANKL/OPG. Bone. (2022) 164:116511. doi: 10.1016/j.bone.2022.116511 35933095

[142] Guo Z Wu J Hu Y Zhou J Li Q Zhang Y. Exogenous iron caused osteocyte apoptosis, increased RANKL production, and stimulated bone resorption through oxidative stress in a murine model. Chem Biol Interact. (2024) 399:111135. doi: 10.1016/j.cbi.2024.111135 38971422

[143] Li Y Huang Z Pan S Feng Y He H Cheng S. Resveratrol alleviates diabetic periodontitis-induced alveolar osteocyte ferroptosis possibly via regulation of SLC7A11/GPX4. Nutrients. (2023) 15:2115. doi: 10.3390/nu15092115 37432277 PMC10181281

[144] Tang Y Su S Yu R Liao C Dong Z Jia C. Unraveling ferroptosis in osteogenic lineages: implications for dysregulated bone remodeling during periodontitis progression. Cell Death Discovery. (2024) 10:195. doi: 10.1038/s41420-024-01969-6 38670955 PMC11053120

[145] Li G Liao C Chen J Wang Z Zhu S Lai J. Targeting the MCP-GPX4/HMGB1 axis for effectively triggering immunogenic ferroptosis in pancreatic ductal adenocarcinoma. Adv Sci (Weinh). (2024) 11:e2308208. doi: 10.1002/advs.202308208 38593415 PMC11151063

[146] Feng Z Meng F Huo F Zhu Y Qin Y Gui Y. Inhibition of ferroptosis rescues M2 macrophages and alleviates arthritis by suppressing the HMGB1/TLR4/STAT3 axis in M1 macrophages. Redox Biol. (2024) 75:103255. doi: 10.1016/j.redox.2024.103255 39029270 PMC11304870

[147] Wei Y Liu W Wang R Chen Y Liu J Guo X. Propionate promotes ferroptosis and apoptosis through mitophagy and ACSL4-mediated ferroptosis elicits anti-leukemia immunity. Free Radic Biol Med. (2024) 213:36–51. doi: 10.1016/j.freeradbiomed.2024.01.005 38215892

[148] Liu J Zhu S Zeng L Li J Klionsky DJ Kroemer G. DCN released from ferroptotic cells ignites AGER-dependent immune responses. Autophagy. (2022) 18:2036–49. doi: 10.1080/15548627.2021.2008692 PMC9397459 34964698

[149] Ru Q Li Y Xie W Ding Y Chen L Xu G. Fighting age-related orthopedic diseases: focusing on ferroptosis. Bone Res. (2023) 11:12. doi: 10.1038/s41413-023-00247-y 36854703 PMC9975200

[150] Das BK Wang L Fujiwara T Zhou J Aykin-Burns N Krager KJ. Transferrin receptor 1-mediated iron uptake regulates bone mass in mice via osteoclast mitochondria and cytoskeleton. Elife. (2022) 11:e73539. doi: 10.7554/eLife.73539 35758636 PMC9352353

[151] Li T Du Y Yao H Zhao B Wang Z Chen R. Isobavachin attenuates osteoclastogenesis and periodontitis-induced bone loss by inhibiting cellular iron accumulation and mitochondrial biogenesis. Biochem Pharmacol. (2024) 224:116202. doi: 10.1016/j.bcp.2024.116202 38615917

[152] Martinon F Burns K Tschopp J. The inflammasome: a molecular platform triggering activation of inflammatory caspases and processing of proIL-beta. Mol Cell. (2002) 10:417–26. doi: 10.1016/s1097-2765(02)00599-3 12191486

[153] Shi J Zhao Y Wang K Shi X Wang Y Huang H. Cleavage of GSDMD by inflammatory caspases determines pyroptotic cell death. Nature. (2015) 526:660–5. doi: 10.1038/nature15514 26375003

[154] Santa Cruz Garcia AB Schnur KP Malik AB Mo GCH. Gasdermin D pores are dynamically regulated by local phosphoinositide circuitry. Nat Commun. (2022) 13:52. doi: 10.1038/s41467-021-27692-9 35013201 PMC8748731

[155] Bertheloot D Latz E Franklin BS. Necroptosis, pyroptosis and apoptosis: an intricate game of cell death. Cell Mol Immunol. (2021) 18:1106–21. doi: 10.1038/s41423-020-00630-3 PMC8008022 33785842

[156] Li X Ji L Men X Chen X Zhi M He S. Pyroptosis in bone loss. Apoptosis. (2023) 28:293–312. doi: 10.1007/s10495-022-01807-z 36645574 PMC9842222

[157] Zhao S Ge C Li Y Chang L Dan Z Tu Y. Desferrioxamine alleviates UHMWPE particle-induced osteoclastic osteolysis by inhibiting caspase-1-dependent pyroptosis in osteocytes. J Biol Eng. (2022) 16:34. doi: 10.1186/s13036-022-00314-8 36482442 PMC9733322

[158] Zhang Y Yan M Shan W Zhang T Shen Y Zhu R. Bisphenol A induces pyroptotic cell death via ROS/NLRP3/Caspase-1 pathway in osteocytes MLO-Y4. Food Chem Toxicol. (2022) 159:112772. doi: 10.1016/j.fct.2021.112772 34929351

[159] Dai SM Nishioka K Yudoh K. Interleukin (IL) 18 stimulates osteoclast formation through synovial T cells in rheumatoid arthritis: comparison with IL1 beta and tumour necrosis factor alpha. Ann Rheum Dis. (2004) 63:1379–86. doi: 10.1136/ard.2003.018481 PMC1754791 15479886

[160] Lei L Sun J Han J Jiang X Wang Z Chen L. Interleukin-17 induces pyroptosis in osteoblasts through the NLRP3 inflammasome pathway *in vitro* . Int Immunopharmacol. (2021) 96:107781. doi: 10.1016/j.intimp.2021.107781 34004438

[161] Chen L Yu H Li Z Wang Y Jin S Yu M. Force-induced Caspase-1-dependent pyroptosis regulates orthodontic tooth movement. Int J Oral Sci. (2024) 16:3. doi: 10.1038/s41368-023-00268-7 38221531 PMC10788340

[162] Li X Men X Ji L Chen X He S Zhang P. NLRP3-mediated periodontal ligament cell pyroptosis promotes root resorption. J Clin Periodontol. (2024) 51:474–86. doi: 10.1111/jcpe.13914 38164052

[163] Yin J Yin Z Lai P Liu X Ma J. Pyroptosis in periprosthetic osteolysis. Biomolecules. (2022) 12:1733. doi: 10.3390/biom12121733 36551161 PMC9775904

[164] Li Y Yuan Y Huang ZX Chen H Lan R Wang Z. GSDME-mediated pyroptosis promotes inflammation and fibrosis in obstructive nephropathy. Cell Death Differ. (2021) 28:2333–50. doi: 10.1038/s41418-021-00755-6 PMC8329275 33664482

[165] Li D Gao Z Li Q Liu X Liu H. Cuproptosis-a potential target for the treatment of osteoporosis. Front Endocrinol (Lausanne). (2023) 14:1135181. doi: 10.3389/fendo.2023.1135181 37214253 PMC10196240

[166] Sun X Yang Y Meng X Li J Liu X Liu H. PANoptosis: Mechanisms, biology, and role in disease. Immunol Rev. (2024) 321:246–62. doi: 10.1111/imr.13279 37823450

